# Non-Coding RNAs and Resistance to Anticancer Drugs in Gastrointestinal Tumors

**DOI:** 10.3389/fonc.2018.00226

**Published:** 2018-06-18

**Authors:** Jens C. Hahne, Nicola Valeri

**Affiliations:** ^1^Division of Molecular Pathology, The Institute of Cancer Research, London, United Kingdom; ^2^Department of Medicine, The Royal Marsden NHS Trust, London, United Kingdom

**Keywords:** non-coding RNA, lncRNA, microRNA, anticancer drugs, gastrointestinal tumor, cancer therapy, resistance

## Abstract

Non-coding RNAs are important regulators of gene expression and transcription. It is well established that impaired non-coding RNA expression especially the one of long non-coding RNAs and microRNAs is involved in a number of pathological conditions including cancer. Non-coding RNAs are responsible for the development of resistance to anticancer treatments as they regulate drug resistance-related genes, affect intracellular drug concentrations, induce alternative signaling pathways, alter drug efficiency *via* blocking cell cycle regulation, and DNA damage response. Furthermore, they can prevent therapeutic-induced cell death and promote epithelial–mesenchymal transition (EMT) and elicit non-cell autonomous mechanisms of resistance. In this review, we summarize the role of non-coding RNAs for different mechanisms resulting in drug resistance (e.g., drug transport, drug metabolism, cell cycle regulation, regulation of apoptotic pathways, cancer stem cells, and EMT) in the context of gastrointestinal cancers.

## Introduction

Gastrointestinal (GI) cancer encompasses a heterogeneous group of tumors that affect the digestive tract system (1). These include cancers of the esophagus, stomach, gallbladder, liver and biliary tract, pancreas, small intestine, colon, rectum, and anus. GI cancer is the most common form of cancer responsible for nearly 25% of all new cancer diagnosis and responsible for most of cancer-related death (around 30% of all cancer-related death) worldwide (2, 3).

Chemotherapy is, alongside with surgery and radiation therapy, one of the main treatments for cancer (4–12). Many chemotherapeutic agents have successfully prolonged overall and progression-free survival of GI cancer patients (13–17). In addition, a better understanding of the biology and mechanism underpinning GI cancer initiation and progression is leading to more personalized treatments. Indeed, identification of well-defined molecular subtypes and/or molecular profiling of somatic mutations offer the opportunity to further optimize the efficacy of treatments through tailored approaches (18–21).

**Figure 1:**
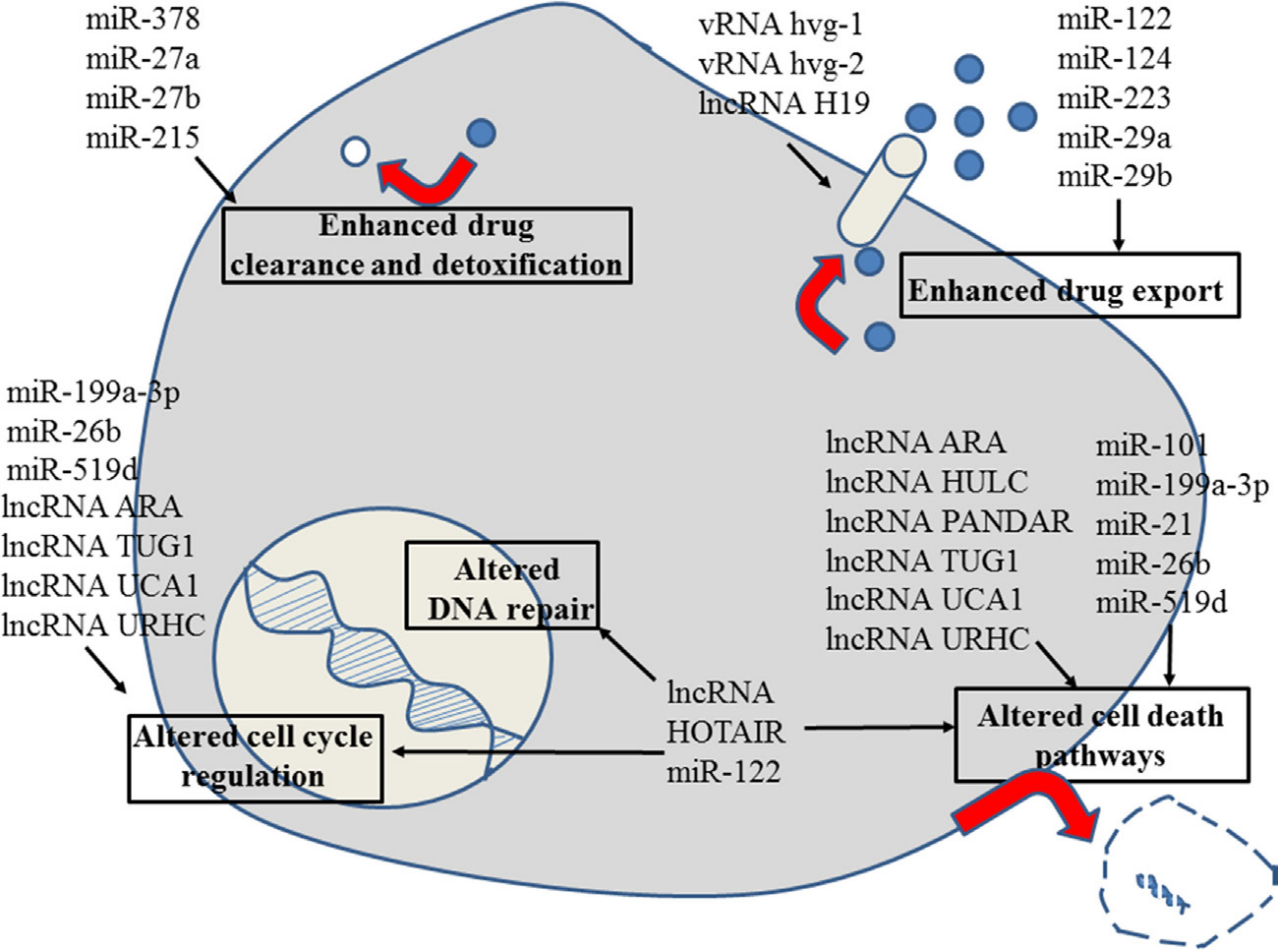
Role of non-coding RNAs for the different reasons that can cause resistance to anticancer drugs in liver cancer. For details about target genes and regulated protein expression by the non-coding RNAs, see text.

**Figure 2:**
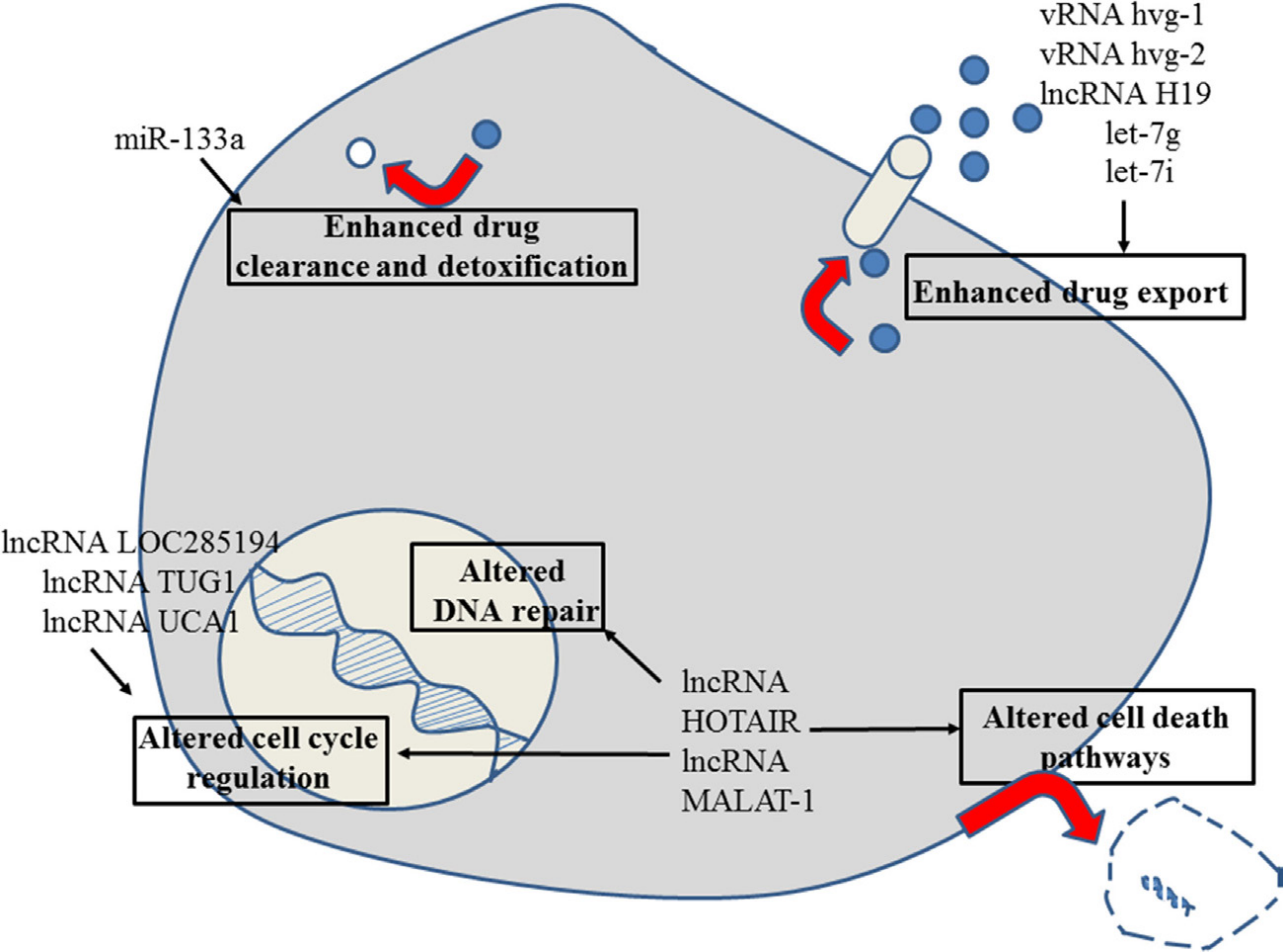
Role of non-coding RNAs for the different reasons that can cause resistance to anticancer drugs in esophageal cancer. For details about target genes and regulated protein expression by the non-coding RNAs, see text.

**Figure 3:**
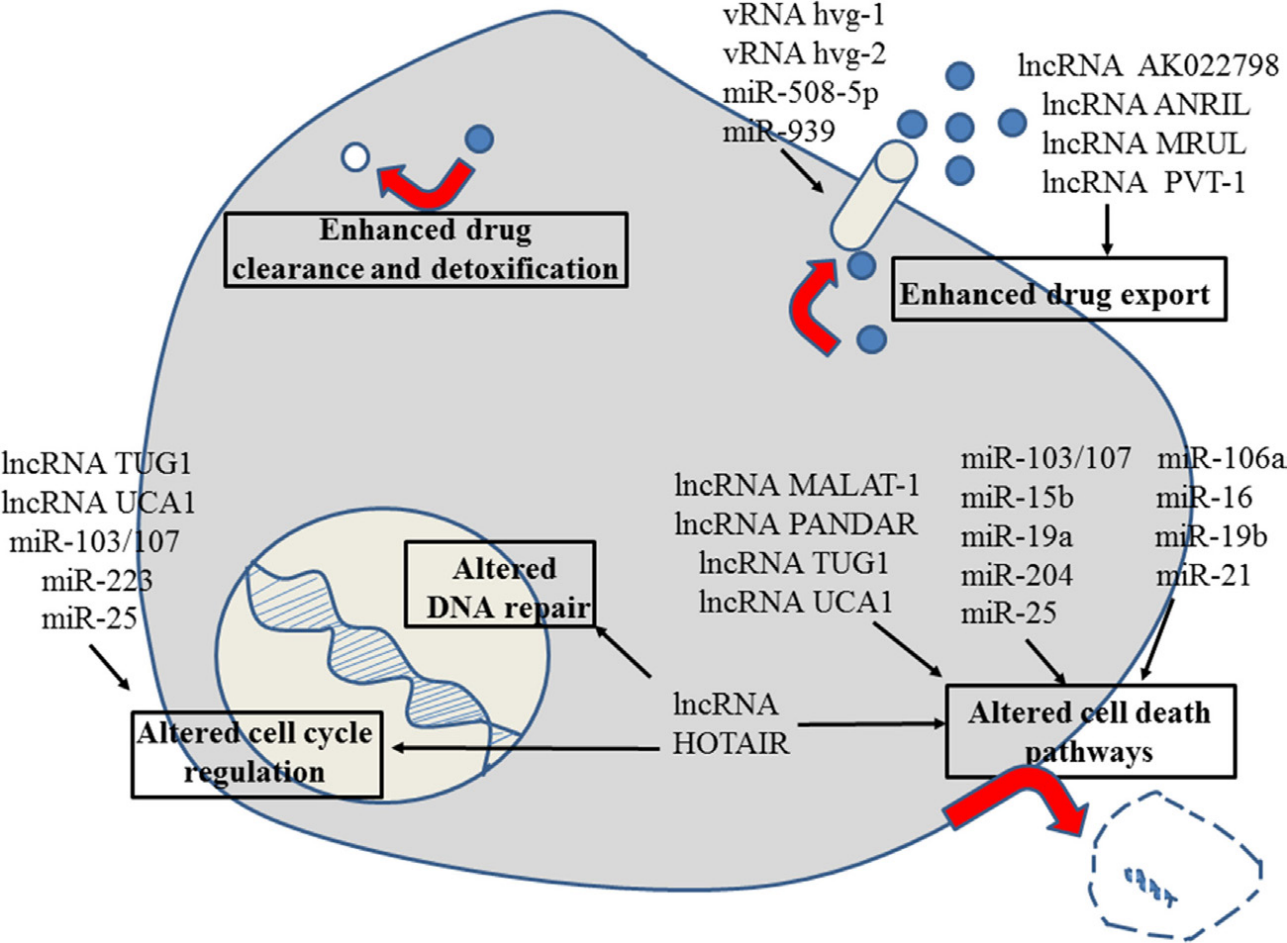
Role of non-coding RNAs for the different reasons that can cause resistance to anticancer drugs in gastric cancer. For details about target genes and regulated protein expression by the non-coding RNAs, see text.

**Figure 4:**
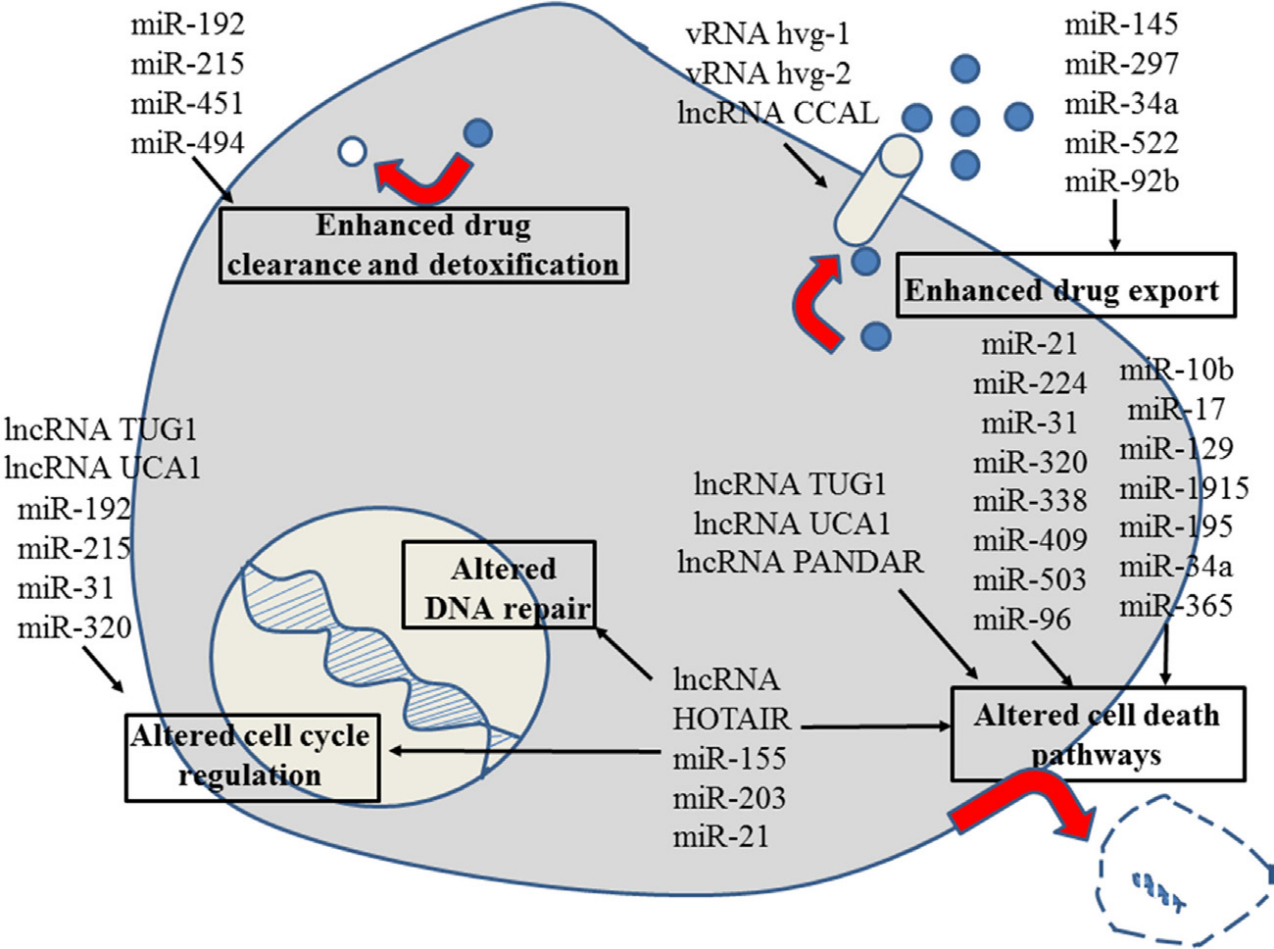
Role of non-coding RNAs for the different reasons that can cause resistance to anticancer drugs in colon and colorectal cancer. For details about target genes and regulated protein expression by the non-coding RNAs, see text.

**Figure 5:**
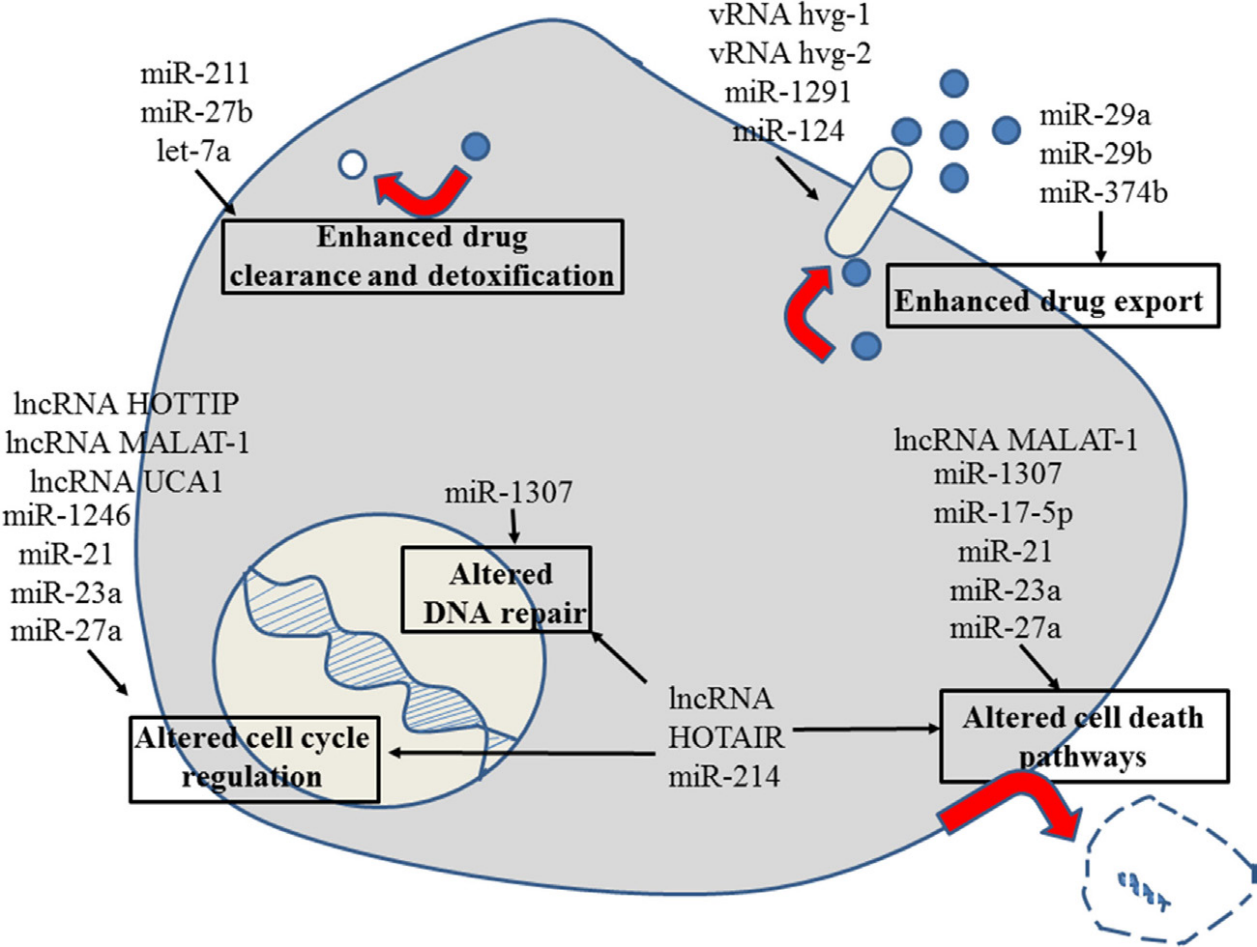
Role of non-coding RNAs for the different reasons that can cause resistance to anticancer drugs in pancreatic cancer. For details about target genes and regulated protein expression by the non-coding RNAs, see text.

**Figure 6:**
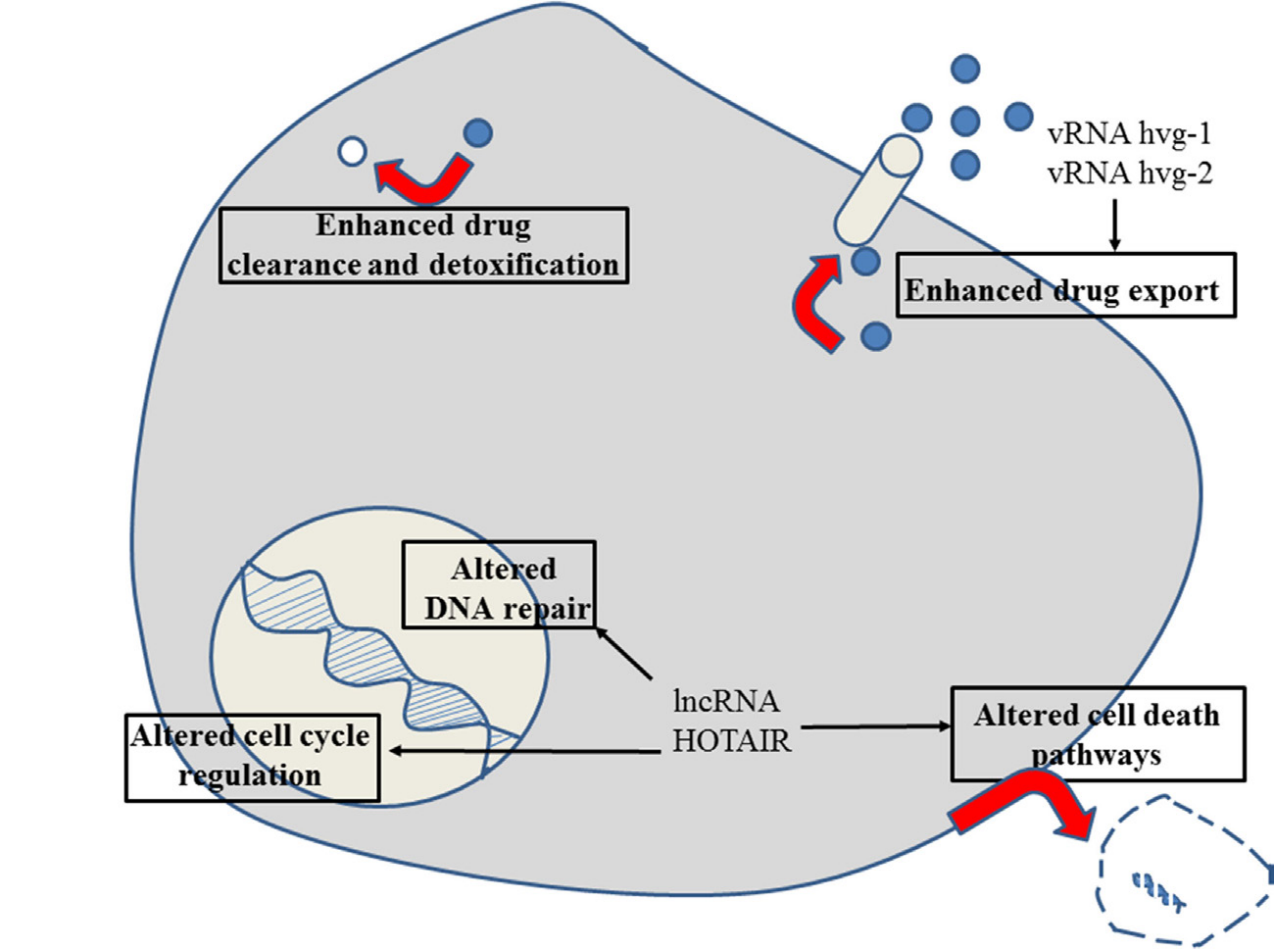
Role of non-coding RNAs for the different reasons that can cause resistance to anticancer drugs in gastrointestinal stromal cancer. For details about target genes and regulated protein expression by the non-coding RNAs, see text.

**Figure 7:**
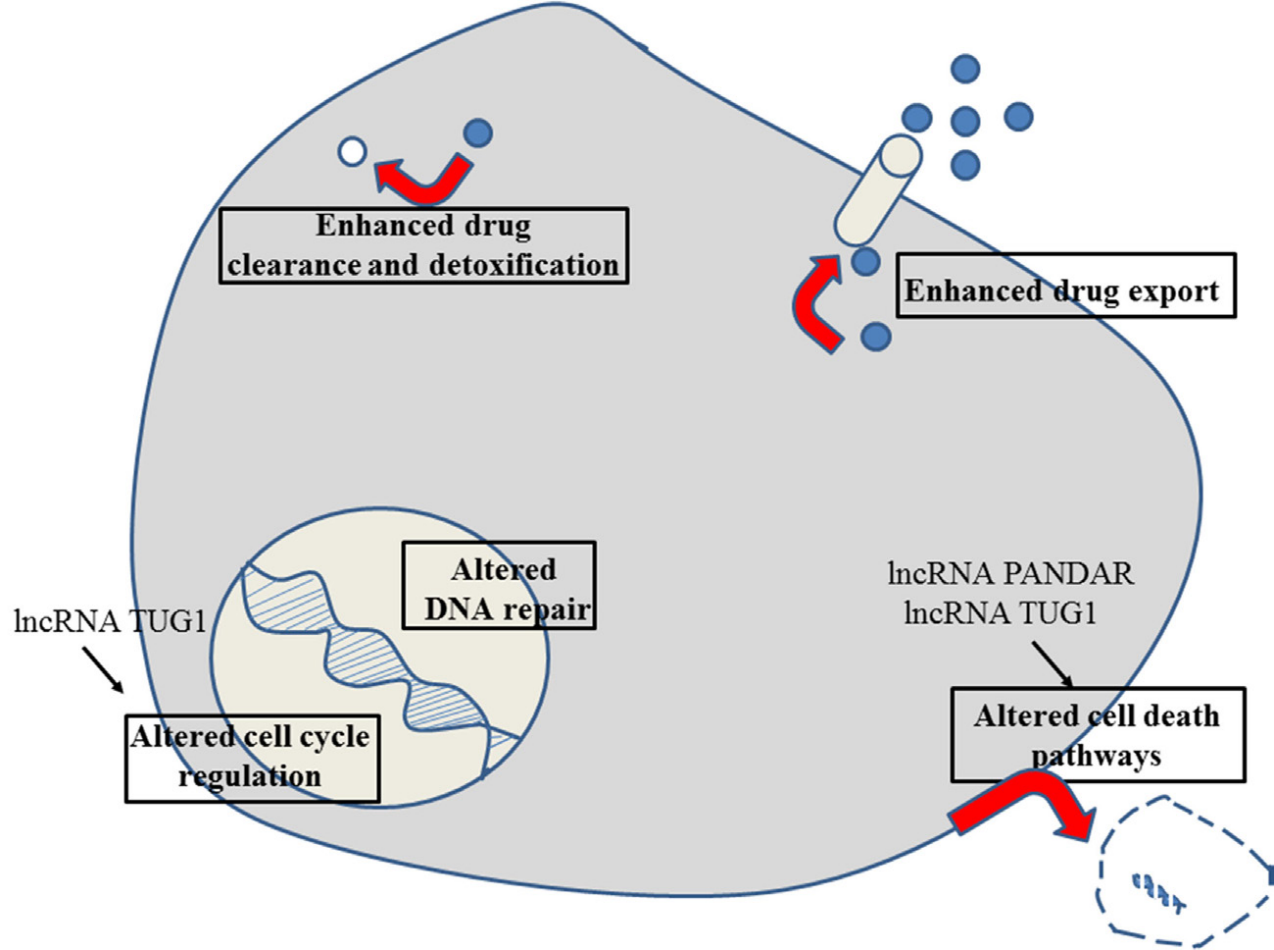
Role of non-coding RNAs for the different reasons that can cause resistance to anticancer drugs in cholangiocarcinoma. For details about target genes and regulated protein expression by the non-coding RNAs, see text.

Despite major improvements in the management of GI cancer patients, resistance to therapies arises almost inevitably at some point during the treatment and chemoresistance is one of the main challenges in cancer therapy (22). Drug resistance can be caused by gene mutations, abnormal DNA repair, alteration in cell cycle regulation, cell death inhibition (mostly caused by deregulated apoptotic signaling pathways), reduced drug efficacy, and enhanced drug clearance (22, 23). Furthermore, the epithelial–mesenchymal transition (EMT) process and the presence of tumor stem cells have been identified as causes of drug resistance (24–27). The complex molecular mechanisms of chemoresistance have not been fully elucidated yet and a better understanding of drivers of primary and secondary resistance to chemotherapy will likely result into improved patients’ survival. Increasing evidence points toward the role of non-coding RNAs as a central hub for treatment resistance. Therefore, this review outlines the role of non-coding RNAs for the different drug resistance mechanisms involved in GI cancer therapy failure. Table 1 summarized the non-coding RNAs discussed in this review; and in Figures 1–7, the role for each of these non-coding RNAs in the context of the different GI tumors is illustrated.

**Table 1:** Overview about non-coding RNAs involved in resistance to anticancer drugs in gastrointestinal tumors.

Non-coding RNA	GI cancer type	Causing drug resistance *via*	Reference
lncRNA AK022798	Gastric cancer	Increasing the expression of *ABCB1* gene	(28)
lncRNA ANRIL	Gastric cancer	Increasing the expression of *MDR1* gene	(29, 30)
lncRNA ARA	Liver cancer	Reduced G2/M cell-cycle arrest; reduced apoptosis rate; deregulation of MAPK-pathway	(31, 32)
lncRNA-ATB	Liver cancer	Increased expression of ZEB1 and ZEB2; induced EMT	(33)
lncRNA CCAL	Colorectal cancer	Increasing the expression of *ABCB1* gene; increased activity of Wnt/β-catenin pathway	(34)
lncRNA H19	Liver cancer esophageal cancer	Upregulation of membrane glycoprotein p95; elevating the expression of *MDR1* gene by increasing promoter methylation; increasing telomere length	(35–37)
lncRNA HOTAIR	Liver cancer Colorectal cancer Pancreatic cancer Gi stromal tumor	Increased expression of PRC2 complex members; genome-wide changes in transcription process due to epigenetic chromatin silencing; downregulation of p21(WAF/CIP1); repression of G1/S cell-cycle arrest; increased proliferation rate; reduced DNA-damage response	(38–41)
lncRNA HOTAIR	Colon cancer Pancreatic cancer Gastric cancer esophageal cancer	Transformation of stem cells into cancer stem cells due to activation of *OCT4, RNF51, CD44*, and *CD133* gene expression; increased activity of Wnt/β-catenin pathway; modulation of chromatin organization leads to reduced efficiency of the mismatch repair system; increased MSI; reduced apoptosis rate; inhibition of the expression of miR-126 and activating the PI3K-AKT-mTOR pathway (in gastric cancer)	(42–48)
lncRNA HOTTTIP	Pancreatic cancer	Increased expression of transcription factor HOX13; cell cycle deregulation	(49, 50)
lncRNA HULC	Liver cancer	Increased activity of Wnt-β-catenin; increased expression of USP22 and SIRT1; reduced expression of miR-6825-5p, miR-6845-5p, miR-6886-3p; increased autophagy pathway	(51)
lncRNA HULC	Gastric cancer	Induced EMT; suppressed apoptosis	(52, 53)
lncRNA LEIGG	Gastric cancer	Induced EMT	(54, 55)
lncRNA linc-ROR	Pancreatic cancer	Inhibition of p53; inhibition of the expression of miR-200 family; increased expression of the transcription factor ZEB1; induced EMT	(56, 57)
lncRNA linc-ROR	Liver cancer	Preventing the binding of miR-145 to pluripotent factors OKT-4, NANOG, and SOX2 resulting in increased expression of these transcription factors necessary for sustain stem cell character	(58, 59)
lncRNA LOC285194	esophageal cancer	Cell-cycle deregulation; blocking non-apoptotic cell death pathway	(60)
lncRNA MALAT-1	esophageal tumor	Binds miR-107 and miR-217; reduced activity of the ATM-CHK2 signaling pathway; reduced cell-cycle arrest and cell death as response to DNA damage; increased expression of transcription factor B-Myb	(61–63)
lncRNA MALAT-1	Pancreatic cancer	Increased expression of cancer stem cell marker CD133; increased expression of pluripotent factors OCT4, NANOG, and SOX2; induced EMT; repression of G2/M cell-cycle arrest; reduced apoptosis rate	(64–66)
lncRNA MALAT-1	Gastric cancer	Sequestering of miR-23b-3p; increased expression of ATG12; increased autophagy	(67)
lncRNA MIR100HG	Colon cancer	Increased activity of Wnt-β-catenin pathway	(68)
lncRNA MRUL	Gastric cancer	Increasing the expression of *MDR1* gene	(69)
lncRNA PANDAR	Gastric cancer Colorectal cancer Hepatocellular cancer cholangiocarcinoma	Interacts with the transcription factor NF-YA resulting in reduced translation of proapoptotic genes—leading to reduced apoptosis rate and increased proliferation	(70–74)
lncRNA PVT1	Gastric cancer esophageal cancer Pancreatic cancer Colon cancer Liver cancer	Induced EMT	(75–77)
lncRNA PVT-1	Gastric cancer	Increasing the expression of *MDR1* gene	(29, 30)
lncRNA TUC338	Hepatocellular cancer	Inhibiting the RASAL-1 pathway	(78)
lncRNA TUG1	esophageal cancer Gastric cancer Colorectal cancer Hepatocellular cancer cholangiocarcinoma	Increasing the expression of *Bc-2* gene; reducing the expression of cyclin-dependent protein kinase, caspase-3, caspase-9, and Bax; decreasing G0/G1 arrest during cell cycle; reducing apoptosis rate; inducing EMT	(79–85)
lncRNA UCA1 (identical with lncRNA CDUR)	Liver cancer Colorectal cancer Pancreatic cancer Gastric cancer esophageal cancer	Sequestering microRNAs (miR-216b in liver cancer; miR-204-5p in colorectal and esophageal cancer; miR-27 in gastric cancer); increase expression of lncRNAs (HULC; H19); increased activity of Wnt-β-catenin pathway; increased activity of PI3K-AKT-mTOR pathway; increased phosphorylation of tumor suppressor retinoblastoma; increased expression of c-myc; increased cell-cycle progression; increased expression of antiapoptotic protein Bcl-2; reduced expression of PARP (in gastric cancer); reduced apoptosis rate. In liver cancer, additional effects: transformation of stem cells into cancer stem cells due to increased c-myc expression; increasing telomere length	(35, 86–96)
lncRNA URHC	Liver cancer	Reduced expression of the tumor suppressor ZAK; increased proliferation rate; reduced apoptosis rate	(97)
lncRNA-34a	Colon cancer	Increased activity of Wnt-β-catenin pathway; increased activity of NOTCH pathway; increasing the self-renewal of cancer stem cells	(98, 99)
miR let-7 family	Pancreatic cancer	Induced EMT	(100)
miR let-7a	Pancreatic tumors	Increased expression of RRM2	(101)
miR let-7g	esophageal cancer	Increased expression of ABCC10	(102)
miR let-7i	esophageal cancer	Increased expression of ABCC10	(102)
miR-100	Colon cancer	Increased activity of Wnt-β-catenin pathway	(68)
miR-101	Liver cancer	Increased expression of EZH2; increased activity of Wnt-β-catenin pathway; increased expression of Mcl-1; reduced apoptosis rate	(103–105)
miR-10b	Colorectal cancer	Increased expression of antiapoptotic protein BIm	(106)
miR-103/107	Gastric cancer	Reduced expression of tumor-suppressor caveolin-1; activation of Ras-p42/p44 MAP pathway; reduced apoptosis rate	(107–109)
miR-106a	Gastric cancer	Reduced expression of FAS; reduced apoptosis rate	(110, 111)
miR-1182	Gastric cancer	Increased expression of hTERT	(112)
miR-122	Liver cancer	Increased expression of ABC proteins; increased expression of cyclin G1; reduced G2/M cell-cycle arrest; reduced DNA repair; reduced apoptosis rate	(113, 114)
miR-124	Pancreatic cancer Liver cancer	Reduced expression of SLC16A1	(115)
miR-125b	Colon cancer	Increased activity of Wnt-β-catenin pathway	(68)
miR-1246	Pancreatic cancer	Reduced expression of cyclin-G2; deregulated cell-cycle	(116)
miR-129	Colorectal cancer	Increased expression of antiapoptotic protein Bcl-2	(117)
miR-1291	Pancreatic cancer	Increased expression of ABCC1	(118)
miR-130b	Liver cancer	Reduce expression of tumor protein 53-induced nuclear protein 1	(119)
miR-1307	Pancreatic cancer	Reduced apoptosis rate	(120)
miR-133a	esophageal cancer	Increased expression of GSTP1	(121)
miR-145	Colon carcinoma	Increased expression of ABCB1	(122)
miR-147	Colon cancer	Induced EMT; increased phosphorylation of AKT; increased activity of PI3K-AKT-mtor pathway; increased activity of TGF-β pathway	(123)
miR-155	Colorectal cancer	Inhibition of MSH2, MSH6, and MLH1	(124)
miR-15b	Gastric cancer	Increased expression of antiapoptotic protein Bcl-2	(125)
miR-16	Gastric cancer	Increased expression of antiapoptotic protein Bcl-2	(125)
mir-17-5p	Colorectal cancer	Reduced expression of PTEN expression; activation of AKT-mtor pathways	(126)
miR-17-5p	Pancreatic cancer	Reduced expression of BIM	(127)
miR-1915	Colon cancer	Increased expression of BCL-2	(128)
miR-192	Colon cancer	Reduced expression of thymidylate synthase; altered cell-cycle control at multiple levels; prevent progression into the S-phase	(129)
miR-193b	Hepatocellular cancer	Increased expression of Mcl-1	(130)
miR-195	Colorectal cancer	Increased expression of antiapoptotic protein Bcl-2L2	(131)
miR-199a-3p	Liver cancer	Reduced G1/S cell-cycle arrest; increased expression of mtor and c-Met; reduced apoptosis rate	(132, 133)
miR-19a	Gastric cancer	Reduced expression of PTEN expression; activation of AKT-mtor pathways	(134)
miR-19b	Gastric cancer	Reduced expression of PTEN expression; activation of AKT-mtor pathways	(134)
miR-200a	Pancreatic cancer	Induced EMT	(100)
miR-200b	Pancreatic cancer	Induced EMT	(100)
miR-200c	Pancreatic cancer	Induced EMT	(100, 135)
miR-203	Colorectal cancer	Reduced expression of ATM; impaired DNA repair; reduced apoptosis rate	(136)
miR-205	Pancreatic cancer	Increased expression of pluripotent factors OKT3, OKT8, and CD44	(137)
miR-21	Colorectal cancer	Inhibition of MSH2 and MSH6; reduced G2/M cell-cycle arrest; reduced apoptosis rate; increasing the number of undifferentiated cancer stem cells	(138, 139)
miR-21	Pancreatic cancer	Reduced cell-cycle arrest; reduced expression of PTEN; activation of AKT-mtor pathway; increased expression of antiapoptotic protein Bcl-2; increased cell proliferation; reduced apoptosis rate	(140, 141)
miR-21	Liver cancer Gastric cancer	Reduced expression of PTEN expression; activation of AKT-mtor pathways	(142–144)
Synergistic action of miR-21 miR-23a miR-27a	Pancreatic cancer	Reduced expression of the tumor suppressors PDCD4, BTG2, and NEDD4L; deregulated cell-cycle; reduced apoptosis rate	(145, 146)
miR-211	Pancreatic tumors	Increased expression of RRM2	(147)
miR-215	Liver cancer	Reduced expression of dihydrofolate reductase; reduced expression of thymidylate synthase	(148)
miR-215	Colon cancer	Reduced expression of thymidylate synthase; altered cell-cycle control at multiple levels; prevent progression into the S-phase	(129)
miR-215	Gastric cancer	Reduced expression of retinoblastoma 1; altered cell-cycle control	(149, 150)
miR-22	P53-mutated colon cancer	Reduced expression of PTEN expression; activation of AKT-mtor pathways	
miR-221	esophageal cancer	Reduced expression of DDK2; activation of Wnt/β-catenin pathway; induced EMT	(151, 152)
miR-223	Liver cancer	Increased expression of ABCB1	
miR-223	Pancreatic cancer	Induced EMT	(153)
miR-223	Gastric cancer	Reduced expression of FBXW7; altered cell-cycle control	(154)
miR-224	Colon cancer	Induced EMT; increased phosphorylation of AKT und ERK; increased activity of PI3K-AKT-mtor pathway; increased activity of ERK pathway; activation of NF-κB; and EGFR dependent pathways	(155)
miR-23a	Microsatellite instable colon cancer	Increased expression of ABCF1	(156)
miR-25	Gastric cancer	Reduced expression of FOXO3a, ERBB2, and FBXW7; cell-cycle deregulation; reduced apoptosis rate	(157–160)
miR-26b	Liver cancer	Increased activation of NF-κB	(161, 162)
miR-27a	Liver cancer	Reduced expression of dihydropyrimidine dehydrogenase	(163)
miR-27b	Liver cancer	Increased expression of CYP1B1; reduced expression of dihydropyrimidine dehydrogenase	(163, 164)
miR-27b	Pancreatic cancer	Reduced expression of CYP3A4—resulting in cyclophosphamide resistance due to missing drug activation	(165)
miR-297	Colorectal cancer	Increased expression of ABCC2	(166)
miR-29a	Pancreatic cancer Liver cancer	Reduced expression of SLC16A1	(115)
miR-29b	Pancreatic cancer Liver cancer	Reduced expression of SLC16A1	(115)
miR-31	Colorectal cancer	Cell-cycle deregulation; reduced apoptosis rate	(167, 168)
miR-320	Colon cancer	Increased expression of SOX4; inhibition of p53 mediated apoptosis; reduced expression of FOXM1 and FOXQ1; cell-cycle deregulation	(169, 170)
miR-338-3p	p53 mutant colorectal cancer	Reduced expression of mtor; increased autophagy; and reduced apoptosis rate	(171)
miR-34a	Colon cancer	Increased expression of antiapoptotic protein Bcl-2	(172)
miR-365	Colon cancer	Increased expression of antiapoptotic protein Bcl-2	(173)
miR-374b	Pancreatic cancer	Increased ATP7A expression	(174)
miR-378	Liver cancer	Increased expression of CYP2E1	(175)
miR-409-3p	Colon cancer	Increased expression of Beclin-1; increased autophagy pathway	(176)
miR-451	Colon cancer	Increasing the self-renewal of cancer stem cells; increased expression of ABCB1	(177)
miR-494	Colon cancer	Reduced expression of dihydropyrimidine dehydrogenase	(178)
miR-503-5p	Colorectal cancer	Reduced expression of apoptotic protein PUMA	(179)
miR-508-5p	Gastric cancer	Increased expression of ABCB1; increased expression of transcription factor ZNRD1	(180)
miR-519d	Liver cancer	Reduced expression of G1-checkpoint CDK inhibitor p21; reduced apoptosis rate	(181)
miR-522	Colon cancer	Increased expression of ABCB5	(182)
miR-92b	Colon cancer	Reduced expression of SLC15A and SLC15A1	(183)
miR-939	Gastric cancer	Increased expression of SLC34A2; activation of Ras/MEK/ERK pathway	(184)
miR-96	Colorectal cancer	Reduced expression of antiapoptotic proteins XIAP and UBE2N	(185)
svRNAb	All GI tumors	Reduced expression of CYP3A4	(186)
vRNA hvg-1	All GI tumors	Transporting drugs away from the target and drug sequestration	(187, 188)
vRNA hvg-2	All GI tumors	Transporting drugs away from the target and drug sequestration	(187, 188)

*GI, gastrointestinal; vRNA, vault RNA; lncRNA, long non-coding RNA; miR, microRNA; EMT, epithelial–mesenchymal transition*.

## Non-Coding RNAs

In human tissues, the amount of non-coding RNAs is more than three times higher compared to the amount of protein-coding RNAs (189). Non-coding RNAs are a large family that includes more than 16 categories of long and short RNA molecules (Table 2); among them transfer RNAs (tRNAs), ribosomal RNAs (rRNAs), small nucleolar RNAs (snoRNAs), endogenous small interfering RNAs (endo-siRNAs), sno-derived RNAs (sdRNAs), transcription initiation RNAs (tiRNAs), miRNA-offset-RNAs (moRNAs), circular RNAs (circRNAs), vault RNAs (vRNAs), microRNAs, small interfering RNAs (siRNAs), small nuclear RNAs (snRNAs), extracellular RNAs (exRNAs), piwi-interacting RNAs (piRNAs), small Cajal body RNAs (scaRNAs), long intergenic non-coding RNAs (lincRNAs), and long non-coding RNAs (lncRNAs), all of which are not coding for known proteins (190–211).

**Table 2:** Overview about the different categories of non-coding RNA molecules.

Name	Biological role
Circular RNA (circRNA)	Involved in forming RNA-protein complex that regulate gene transcription; involved in regulating gene expression at posttranscriptional level by acting as miRNA sponge
Endogenous small interfering RNA (endo-siRNA)	Involved in repression of transposable elements, chromatin organization as well as gene regulation at transcriptional and posttranscriptional level
Extracellular RNA (exRNA)	Involved in intercellular communication and cell regulation
Long intergenic non-coding RNA (lincRNA)	Involved in gene expression *via* directing chromatin-modification complexes to specific target regions; lincRNAs located in the cytoplasm function as scaffold to bring together proteins and other RNA categories (especially mRNAs and miRNAs)
Long non-coding RNA (lncRNA)	Involved in regulation of gene expression *via* binding to chromatin regulatory proteins; involved in regulating gene expression at posttranscriptional level by acting as microRNA decoys; some lncRNAs are processed into microRNAs
MicroRNA	Involved in fine tuning cell homeostasis by controlling gene expression at posttranscriptional level
miRNA-offset-RNA (moRNA)	Unknown
piwi-interacting RNA (piRNA)	Involved in maintain germline integrity by repressing transposable elements; involved in mRNA deadenylation
Ribosomal RNA (rRNA)	Component of the ribosomes; involved in protein synthesis
Small Cajal body RNA (scaRNA)	Component of the Cajal bodies; involved in the biogenesis of small nuclear ribonucleoproteins and by this influence splicing of pre-mRNAs
Small interfering RNA (siRNA)	Involved in RNA interference pathway as part of antiviral defense
Small nuclear RNA (snRNA)	Component of the spliceosome; involved in splicing of pre-mRNAs during posttranscriptional modifications
Small nucleolar RNA (snoRNA)	Component of the Cajal bodies; involved in modification and processing of snRNA, rRNA and tRNA precursors as well as in mRNA editing
sno-derived RNA (sdRNA)	Component of the Cajal bodies; involved in alternative splicing of mRNAs; some sdRNAs control gene expression at posttranscriptional level
Transcription initiation RNA (tiRNA)	Involved in regulation of RNA polymerase II dependent transcription
Transfer RNA (tRNA)	Involved in transporting amino acids to the ribosomes during translation
Vault RNA (vRNA)	Component of the vaults (large ribonucleoprotein complexes in cytoplasm); unknown function

Long non-coding RNAs (lncRNAs) and microRNAs are the most studied non-coding RNAs playing a role in anticancer drug resistance and will be covered in this review.

LncRNAs are composed of more than 200 nucleotides. They are important regulators during development and pathological processes (212–216). LncRNAs are pivotal in regulating gene expression by binding to chromatin regulatory proteins and they are able to alter chromatin modification as well as transcriptional or posttranscriptional gene regulation by interacting with other RNAs and proteins (217–219). Recently, a crosstalk and strong linkage between lncRNA and microRNAs has been identified (220). It has been shown that lncRNA stability can be reduced by interaction with specific microRNAs and, *vice versa*, lncRNAs act as microRNA decoys sequestering microRNAs from the intracellular cytosol and leading to reexpression of microRNA target genes (220). Furthermore, lncRNAs can promote gene expression by competing with microRNAs for specific binding sites in the non-coding regions of mRNAs and prevent the transcriptional repression caused by microRNAs (220). Interestingly some lncRNAs can be processed into microRNAs (220) suggesting a plastic interaction among different classes of non-coding RNAs.

MicroRNAs are short RNA transcripts of 18–24 nucleotides. They are responsible for fine tuning cell homeostasis by controlling gene expression at posttranscriptional level (221–223). Due to the fact that each microRNAs can have several target mRNAs, the interaction of one microRNA with various target mRNAs results in direct deregulation of different target proteins acting simultaneously in regulation of diverse cellular pathways (224, 225). Therefore, variation in microRNA expression can result in reduced mRNA levels ultimately resulting in changes in protein levels within the cell (225, 226). MicroRNAs expression patterns are tissue specific (227) and often define the physiological status of the cell (228). Strong clinical and preclincial evidence suggests that microRNA aberrant expression plays a role in several diseases including cancer, infectious, neurodegenerative, and immune-related diseases (229–240). Analysis of microRNA expression patterns represents a promising tool for cancer diagnosis, prognosis and treatment prediction. MicroRNAs have been extensively studied in monitoring treatment resistance in consideration of their high stability in tissues and body fluids. In blood, microRNAs are included in RNA-binding multiprotein complexes and/or exosomes and their short length makes microRNAs less prone to degradation and improves their stability under different sample storage conditions in blood (224, 230, 236, 240).

## General Principles of Drug Resistance

Drug resistance is classified into intrinsic and acquired. Primary drug resistance is pre-existing and renders cancer cells immune against the therapy from the very beginning. In contrast, acquired (secondary) drug resistance develops during therapy due to adaptive processes of the tumor (22, 241–244). Different mechanisms are involved in primary and acquired drug resistance and relate to non-coding RNAs dysregulation.

### Deregulation of Proteins Involved in Drug Metabolism

One reason for drug resistance can be found on the level of drug transport. Reduced influx or increased efflux of chemotherapeutics result in lower intracellular drug concentrations and promotes therapy failure (241). Altered drug metabolism is another possible cause for drug resistance. Drug metabolism is a complex pathway composed of multiple proteins for detoxification of foreign compounds (e.g., chemotherapeutics) normally neither produced nor present in a cell (245). This pathway can be subdivided into modification (phase I reaction), conjugation (phase II reaction), and excretion (phase III reaction) (246). Several drug-metabolizing enzymes, especially members of the cytochrome P450 family, together with drug transporters increase the polarity of the drugs during phase I (247, 248). In the following phase II, the polarity of the drugs is further increased by conjugation reactions (249, 250). Finally, in phase III the resulting drug metabolites are exported by transmembrane transporter like ATP-binding cassette (ABC) proteins and solute carrier (SLC) transport proteins (251–254).

The vaults are known to contribute to drug resistance by transporting drugs away from their intracellular targets and vaults are involved in drug sequestration (187). The vRNAs hvg-1 and hvg-2 that are present in the vaults (Table 2) interact with drugs *via* specific binding sites (188). In agreement with their role in regard to drug resistance, the number of vaults is increased in cancer patients who developed resistance under chemotherapy (187). In addition, the vRNAs are producing several small RNAs among them is svRNAb which downregulates the key enzyme in drug metabolism CYP3A4 and accounts so for multidrug resistance in GI cancers (186).

Furthermore, lncRNA H19 was identified as another non-coding RNA involved in drug resistance. The oncogenic potential of lncRNA H19 was demonstrated in different tumor types (e.g., liver and esophageal cancer) and overexpression of lncRNA H19 was observed in parallel with upregulation of the membrane glycoprotein p95 in multidrug-resistant tumors (36, 37). In liver tumor cells, resistant to doxorubicin, etoposide, paclitaxel, and vincristine lncRNA H19 expression was increased (36). LncRNA H19 participates in the regulation of *MDR1* gene (also known as *ABCB1* gene) expression and modulates the drug transport out of the cell (36). *In vitro* models of hepatocellular carcinoma suggest that lncRNA H19 can alter *MDR1* promoter methylation and, in doing so, increases the transcription of P-glycoprotein (36).

Similarly, in gastric cancer, MDR-related and upregulated lncRNA (lncRNA MRUL) acts as an enhancer for transcription of P-glycoprotein (MDR1) (69) increasing the number of transmembrane transporters on the tumor cell membrane and fosters the drug export (69). As we described above, different non-coding RNAs can merge onto the same pathway: this is the case of lncRNA AK022798 whose expression is induced by NOTCH-1 overexpression during gastric cancer progression (28). LncRNA AK022798 in turn upregulates the expression of P-glycoprotein and is responsible for increased cisplatin resistance in gastric cancer patients (28). Similarly, in cisplatin and 5-fluorouracil-resistant gastric cancer patients the expression of lncRNA plasmacytoma variant translocation 1 (PVT-1) and lncRNA ANRIL (antisense to CDKN2B locus) are also increased and these non-coding RNAs promote MDR1 upregulation and drug resistance (29, 30).

Non-coding RNA dysregulation is tissue specific, indeed Wnt-β-catenin pathway activation triggers the expression of a different lncRNA, colorectal cancer-associated lncRNA (CCAL). The effect on phenotype is the same as in other cancers given CCAL in turn upregulates P-glycoprotein expression and causing chemotherapy resistance (34).

Additional to the regulation *via* lncRNAs ABC transporter expression levels are also controlled by miRNAs (255, 256).

In colon cancer, P-glycoprotein expression was found to be directly deregulated at posttranscriptional level by binding of miR-145 to the 3′-UTR of the *MDR1* gene transcript (122). Downregulation of miR-145 results in increased ABCB1 protein level (122). Analogously miR-297 binds to the 3′-UTR of ABCC2 mRNA and supresses the expression of ABCC2 transporter (166). In chemoresistant colorectal carcinoma, miR-297 is often downregulated and consequently ABCC2 is expressed on a higher level compared to the surrounding colon tissue (166). Interestingly, *in vitro* and *in vivo* models suggest that resistance to vincristine and oxaliplatin could be overcome by restoring miR-297 expression in therapy-resistant cells (166). Virtually expression of all the transporters can be affected by microRNA dysregulation; ABCB5 transporter is highly expressed in colon cancer cell lines with downregulated miR-522 expression and renders these cells resistant to doxorubicin treatment (182). miR-522 binds to the ABCB5 mRNA 3′-UTR and overexpression of miR-522 reverse chemoresistance to doxorubicin (182). Similarly, 5-fluorouracil resistance in microsatellite instable colon cancer [caused by deregulated miR-21 or miR-155 (124, 138) as mentioned in detail later] can be enhanced by downregulation of miR-23a resulting in higher expression of the direct target ABCF1 (156).

Similar examples exist across the board: in gastric cancer for example, downregulation of miR-508-5p was identified as a reason for multidrug resistance (180). miR-508-5p represses the expression of P-glycoprotein and the transcription factor zinc ribbon domain-containing 1 (ZNRD1) that is an important factor for *MDR1* gene translation (180). Loss of miR-508-5p decreased drug sensitivity in gastric cancer *in vitro* and *in vivo*, whereas ectopic expression of miR-508-5p overcomes drug resistance (180).

In pancreatic cancer cell lines, expression of the transporter ABCC1 is controlled by miR-1291 binding to the 3′-UTR (118). miR-1291 is often downregulated in pancreatic cancer resulting in an increased expression of ABCC1 that finally leads to higher efflux rate of toxic substances (257, 258). This is the reason for resistance to many chemotherapeutics, such as anthracyclines (e.g., doxorubicin), platinum derivates, and the folate antagonist methotrexate (257, 258). Another transporter, called ATP7A (ATPase Cu^2+^ transporting alpha polypeptide), is upregulated in *in vitro* models of resistant pancreatic tumors due to decreased expression of miR-374b (174) and increased ATP7A protein expression is at least partially responsible for cisplatin resistance in pancreatic cancer model systems (174).

Downregulation of miR-122 in liver tumors results in high expression of ABC transporter proteins and causes increased drug export of doxorubicin in liver cancer patients (114). Similarly, ABCB1 transporter expression is upregulated in hepatocellular cancer cells when the posttranscriptional regulator miR-223 is downregulated and the result is again resistance to doxorubicin treatment (259).

Downregulation of microRNAs let-7g and let-7i results in increased expression of ABCC10 that in turn is responsible for resistance to cisplatin therapy in esophageal cancer patients (102).

An important barrier for oral anticancer drugs is represented by intestinal epithelial cells of the GI tract (256, 260). The absorption of most nutrient components as well as drugs is related to a variety of influx transporters such as members of the SLC transporter family (256). The expression pattern of the SLC transporter varied according to the differentiation status of intestinal epithelial cells which is controlled by microRNAs (261). Therefore, changes in the expression level of microRNAs have most probably an important influence on the drug uptake rate (261). Up to now the role of microRNAs for the expression level of SLC transporter have been studied only in cell culture models for colon carcinoma, liver, pancreatic, and gastric tumors (115, 183). In colon cancer cells, expression of miR-92b reduces the amount of SLC15A and SLC15A1 transporter resulting in decreased drug absorption (183). In the context of liver and pancreatic tumors miR-29a, miR-29b, and miR-124 target SLC16A1 and reduce the expression of this transporter (115). Recently, it was shown that miR-939 targets direct SLC34A2 in gastric cancer (184). In 5-fluorouracil-resistant gastric cancer, miR-939 is downregulated and results in increased expression level of SLC34A2. The transport protein SLC34A2 acts as mediator of miR-939 and activates the Ras/MEK/extracellular signal-regulated kinase (ERK) pathway which is known to be deregulated often in cancer and to cause resistance to chemotherapy (184). In *in vitro* models of gastric cancer, overexpression of miR-939 strongly decreased MEK1/2 phosphorylation as well as Raf-1 level, whereas SLC34A2 restoration rescued these effects (184).

Also for some drug-metabolizing enzymes posttranscriptional regulations by miRNAs have been proven (256, 262, 263). Due to their pivotal role in maintaining chemical and functional homeostasis of cells, cytochrome P450 enzymes are strictly controlled. Under physiological conditions, cytochrome P450 enzymes are involved in the regulation of endogenous molecules like bile acids and steroids and under pathological conditions in the case of chemotherapy these enzymes are important in regard to drug metabolism. Deregulated expression of cytochrome P450 enzymes is linked to drug resistance and therapy failure (264).

For example, miR-378 targets mRNA coding for CYP2E1 and reduces the expression level of CYP2E1 protein in cell culture models of liver tumors (175, 265). In liver cancer patients, CYP2E1 expression is increased while miR-378 is downregulated (175, 265). Also, a direct regulation of CYP1B1 by miR-27b was demonstrated in hepatocellular cancer cell lines (164). Decreased expression of miR-27b results in high expression level of CYP1B1 and renders by this liver tumor resistant to docetaxel treatment (164).

In pancreatic cancer cells, overexpression of miR-27b leads to downregulation of CYP3A4 protein and results in drug resistance to cyclophosphamide because CYP3A4 is necessary for drug activation (165). MicroRNA-based regulation of enzymes involved in phase II reactions are less analyzed but nevertheless, in the context of esophageal cancer, regulation of glutathione S-transferase P1 (GSTP1) was found to be regulated by miR-133a (121). Reduced expression of the tumor suppressor miR-133a resulted in increased level of GSTP1 protein (121). In phase II detoxification reactions—including inactivation of platinum derivates and alkylating reagents—GSTP1 catalyses the addition of glutathione to the drug activated during phase I reactions with electrophiles (249, 250).

A more specific influence of non-coding RNAs on drug metabolism was demonstrated for 5-fluorouracil in liver and colon tumors (163, 178). Dihydropyrimidine dehydrogenase, an important enzyme in 5-fluorouracil metabolism, is repressed by miR-494 in colon tumors and by miR-27a as well as miR-27b in liver cancer (163, 178). The fact that the translation of one and the same enzyme in two different tissues is under the control of different miRNAs underlines the tissue-specific regulation and fine-tuning of protein expression that is exerted by miRNAs.

In liver cancer, the translation of two of the most important targets of chemotherapeutic agents, dihydrofolate reductase and thymidylate synthase, are repressed by upregulation of miR-215 (148). Reduced expression of dihydrofolate reductase and thymidylate synthase leads to the development of insensitivity to doxorubicin treatment (148).

Thymidylate synthase is the target of 5-fluoruracil therapy and this enzyme is downregulated by increased expression of miR-192 and miR-215 in colon cancer patients (129). In this case, altered microRNA expression results in down-modulation of the drug target and leads to therapy failure. In addition, miR-192 and miR-215 alter the cell-cycle control at multiple levels and prevent progression into the S-phase leading to 5-fluorouracil resistance (129).

A similar case was observed in pancreatic tumors where ribonucleotide reductase regulatory subunit M2 (RRM2) the target of gemcitabine is under direct control of miR-211 and let-7a (101, 147). Decreased expression of miR-211 and let-7a results in higher RRM2 protein level and renders the tumors resistant to gemcitabine (101, 147).

### Deregulation of Cell-Cycle, DNA Repair Pathways and Alteration in Death Pathways

Impaired cell cycle regulation and alteration of cell death pathways are common causes of drug resistance (243, 266). Increased cell cycle progression and reduced cell death rate lead to accumulation of mutations and uncontrolled cell proliferation, a hallmark of tumor cells (267). Errors in the DNA-damage response program pathways [nuclear excision repair (NER), base excision repair (BER), and DNA mismatch repair (MMR)] play an important role in cancer progression and chemoresistance (268–271). A complex interaction interplay exists between non-coding RNAs and the DNA-damage pathways: on one hand the DNA-damage pathway induces the expression of several non-coding RNAs especially of microRNAs and on the other hand non-coding RNAs regulate directly the expression of several genes involved in DNA-damage pathway. This interaction is cell type specific and dependent on the intensity and nature of DNA damage (272–276).

LncRNA HOX transcript antisense RNA (HOTAIR) is highly expressed in a broad variety of solid tumors including liver, colorectal, pancreatic, and GI stromal tumors (39, 40, 277). LncRNA HOTAIR reprograms chromatin organization together with the polycomb repressive complex PRC2 (40). Upregulation of lncRNA HOTAIR results in higher expression level of members of the PRC2 complex (SUZ12, EZH2, and H3K27me3) (40). Therefore, increased lncRNA HOTAIR expression is associated with a genome-wide reprogramming *via* PRC2 mediated epigenetic silencing of chromatin (40). In addition, lncRNA HOTAIR downregulates cyclin-dependent kinase inhibitor 1 [p21(WAF/CIP1)] (41) causing the loss of an important regulator of the G_1_ and S phase progression (38, 278, 279). Due to the fact that p21(WAF/CIP1) represents a major target of p53 activity DNA damage in lncRNA HOTAIR expressing tumor cells don’t go into cell cycle arrest and this promote cisplatin resistance (38, 41, 278, 279).

In esophageal, gastric, colorectal, and hepatocellular cancer as well as cholangiocarcinomas, lncRNA taurine-upregulated gene 1 (TUG1) is involved in causing resistance to chemotherapy (79–85). In tumor tissue, lncRNA TUG1 is upregulated and promotes cell growth by increased transcription of the *Bcl-2* gene and epigenetic silencing of cyclin-dependent protein kinase inhibitors (p15, p16, p21, p27, and p57) and proapoptotic genes (caspase-3, caspase-9, and Bax) (79–85). Therefore, lncRNA TUG1 is an excellent example for the fact that non-coding RNAs target simultaneously the expression of different genes; beside increasing the expression level of the antiapoptotic protein Bcl-2, expression of key players in the caspase-mediated apoptosis pathway are inhibited together with different cyclin-dependent protein kinase inhibitors. This results in decreasing the G0/G1 arrest during cell cycle and reduces the apoptosis rate of the tumor cells. Most probably lncRNA TUG1 has also a role in the EMT (83, 85) that increases resistance to drug treatments further as outlined in detail below.

Also, the lncRNA promoter of CDKN1A antisense DNA damage-activated RNA (PANDAR) is often deregulated in different GI tumors like gastric, colorectal, and hepatocellular cancer as well as cholangiocarcinoma (71–74). In all these tumors, upregulation of lncRNA PANDAR results in increased proliferation rate and reduced apoptosis (71–74). LncRNA PANDAR interacts with the transcription factor NF-YA, an important regulator for transcription of proapoptotic genes (70). This interaction between lncRNA PANDAR and NF-YA results in decreased expression of proapoptotic genes and eventually leads to drug resistance (71–74).

LncRNA urothelial carcinoma associated 1 (UCA1) mediates resistance to doxorubicin treatment in gastric cancer (94). In *in vitro* systems, knockdown of lncRNA UCA1 overcomes the doxorubicin resistance due to an increased expression of PARP and reduced expression of Bcl-2 resulting in higher apoptosis rate (94).

Furthermore, it was shown that lncRNA UCA1 sequesters miR-204-5p in colorectal cancer and reduces the level of this microRNA in cancer cells (90). The consequence is enhanced cell proliferation and 5-fluorouracil resistance (90).

Another example of non-coding RNAs influencing cell-cycle is lncRNA adriamycin resistance associated (ARA) (31, 32). LncRNA ARA was found to be overexpressed in doxorubicin-resistant liver cancer cell lines compared to the parental cell lines (31). Downregulation of lncRNA ARA results in cell-cycle arrest in G2/M phase, suppressed proliferation, increased apoptotic cell death and, as expected, a reduced resistance against doxorubicin (31, 32). Furthermore, lncRNA ARA is involved in the regulation of multiple signaling pathways including the MAPK-pathway (31, 32). Beside lncRNA ARA the lncRNA upregulated in hepatocellular carcinoma (URHC) is found among the most upregulated lncRNAs in hepatocellular carcinoma. One target of lncRNA URHC is the tumor-suppressor ZAK (97). Downregulation of ZAK *via* lncRNA URHC results in increased cell proliferation and inhibits apoptosis (97).

In pancreatic cancer, lncRNA HOXA transcript at the distal tip (HOTTIP) upregulates the homeobox-transcription factor HOX13 resulting in deregulation of the cell cycle as well as gemcitabine resistance (49, 50).

Downregulation of lncRNA LOC285194 in esophageal cancer results in resistance to chemoradiotherapy (radiation in combination with platinum- or paclitaxel-based chemotherapy) by influencing cell-cycle progression and non-apoptotic cell death pathway *via* regulating VEGF receptor 1 (60).

In contrast, lncRNA metastasis-associated lung adenocarcinoma transcript-1 (MALAT-1) is strongly overexpressed in esophageal tumor tissue and binds miR-107 and miR-217 (62, 63). miR-107 and miR-217 decoy translates in reduced activity of the ATM-CHK2 signaling pathway leading to reduced cell-cycle arrest and cell death as response to DNA damage (61, 63) and overexpression of the transcription factor B-Myb—an important regulator for G1/S and G2/M cell-cycle progression and cell survival (62, 63).

In addition, several microRNAs have been identified as regulators for cell cycle progression and induction of cell death pathways. Therefore, deregulated microRNA expression pattern is often a reason for drug resistance in GI tumors.

Colorectal cancers with upregulated mir-203 are resistant to oxaliplatin (136). Failure of oxaliplatin therapy is caused by miR-203 mediated downregulation of the important mediator protein for DNA damage response ATM (136). As reaction to DNA damage, ATM induces the expression of DNA repair proteins, interrupts the cell cycle, and induces cell death in the case of extended DNA damage (280). Oxaliplatin resistance can also be caused by upregulation of miR-503-5p in colorectal cancer (179). Increased expression of miR-503-5p results in downregulation of the apoptotic protein p53 upregulated modulator of apoptosis (PUMA) and leads to resistance to oxaliplatin-induced apoptosis (179). In colon cancer tissues, downregulation of miR-320 is linked to resistance to 5-fluorouracil therapy (169). Among the targets for miR-320 is the transcription factor SOX4 which is involved in inhibition of p53-mediated apoptosis as well as the cell cycle regulators FOXM1 and FOXQ1 both known to have oncogenic potential (169, 170).

In colorectal cancer cells, miR-21 overexpression results in inhibition of the MMR proteins MSH2 and MSH6, two important proteins for DNA damage recognition and repair (138). Inhibition of MSH2 and MSH6 leads to reduced G2/M cell-cycle arrest caused by 5-fluorouracil induced DNA damage and lower apoptosis rate *in vitro* and *in vivo* (138). Therefore, miR-21 overexpression reduces the therapeutic efficacy of 5-fluorouracil-based chemotherapy in colorectal cancer treatment (138). Furthermore, it was proven that the core mismatch repair proteins MSH2, MSH6, and MLH1 are also downregulated by miR-155 potentially contributing to drug resistance (124). According to another study, 5-fluorouracil resistance in colorectal cancer cells can also be mediated by increased expression of miR-31 causing cell cycle deregulation and reduced apoptosis rate (167, 168). Efficacy of 5-fluorouracil treatment in colorectal cancer patients can also be limited due to upregulation of antiapoptotic proteins like X-linked inhibitor of apoptosis (XIAP) and ubiquitin-conjugating enzyme E2N (UBE2N) as a consequence of decreased miR-96 expression (185) or due to upregulation of the antiapoptotic proteins Bcl-2, Bcl-2-like protein 11 (BIM), or Bcl-2-like protein 2 (Bcl2L2) by reduced expression of miR-129, miR-10b, or miR-195, respectively (106, 117, 131). In other colon cancer studies, reduced expression levels of miR-365, miR-1915, and miR-34a have been described as reason for increased expression of BCL-2 (128, 172, 173).

Increased Bcl-2 expression has been identified as a reason for resistance to 5-fluorouracil in other GI tumors, too, but the posttranscriptional regulation of mRNA coding for Bcl-2 is under the control of different miRNAs; e.g., in gastric cancer diminished expression of miR-204 is the reason (281). According to another study upregulation of Bcl-2 is caused by lower miR-15b and miR-16 expression level and leads to drug resistance in gastric cancer cells due to reduced apoptosis (125). miR-25 overexpression was related to cisplatin resistance in gastric cancer cells (160). miR-25 targets directly mRNAs coding for tumor suppressors like FOXO3a, ERBB2, and F-box/WD repeat-containing protein 7 (FBXW7) (157–160). All these proteins are involved in cell cycle regulation and apoptosis (160, 282, 283). Upregulation of miR-223 targets FBXW7 and leads to cell-cycle deregulation and cisplatin resistance in gastric tumors (154). Furthermore, upregulation of miR-103/107 results in decreased expression of caveolin-1 in gastric cancer cells (109). The tumor suppressor caveolin-1 is a counter regulator for the Ras-p42/p44 MAP kinase pathway and due to the downregulation by miR-103/107 increased activity of the Ras-p42/44 Map kinase pathway results in increased cell cycle progression and reduced cell death (107, 108). In gastric cancer, increased cell cycle progression is also caused by increased expression of miR-215 resulting in reduced expression of the tumor suppressor retinoblastoma 1, an important cell cycle regulator (149, 150). Upregulation of miR-106a targets FAS and inhibits the extrinsic apoptotic pathway in gastric cancer (110, 111). In turn, reduced amount of FAS leads to increased cell proliferation, reduced apoptosis rate, and drug resistance (110, 111).

Overexpression of miR-21 inhibits cell cycle arrest resulting in increased cell proliferation, reduced apoptotic rate, gemcitabine, and 5-fluorouracil resistance in pancreatic cancer (284–286). Similarly, in other pancreatic cancer studies, miR-21 overexpression results in reduced level of PTEN and Bcl-2 leading to activation of AKT-mTOR pathway, reduced apoptosis, and resistance against gemcitabine treatment (140, 141). Increased expression of miR-214 represses directly ING4 in pancreatic tumor (287). This impairs cell-cycle arrest, DNA repair as well as apoptosis and results in resistance to gemcitabine treatment (287). The expression of the important proapoptotic protein BIM is reduced by miR-17-5p in pancreatic cancer and results in decreased apoptotic rate leading to resistance to gemcitabine treatment (127). Therapy failure is also caused by the repression of a tumor suppressor network involved in cell cycle and apoptosis regulation composed of PDCD4, BTG2 and NEDD4L by the combined action of miR-21, miR-23a, and miR-27a (145, 146). Furthermore, overexpression of miR-1246 results in decreased expression of cyclin-G2 and impairs the cell cycle regulation resulting in resistance to gemcitabine (116). Recently, miR-1307 was identified to be responsible for FOLFIRINOX resistance in pancreatic cancer (120). miR-1307 is upregulated in *in vitro* models of FOLFIRINOX-resistant pancreatic cancer as well as in patient derived material compared to the surrounding tissue (120). Reduced apoptosis rate and an extended acceptance of DNA damage seem to be the consequence of higher miR-1307 expression (120).

In hepatocellular carcinoma, the liver specific miR-122 is downregulated and as consequence the expression of the target gene *CCNG1* is increased (113). High level of cyclin G1 protein is found in several human tumors and results in reduced cell cycle control in the G2/M phase and modulation of p53 activity (113, 114). This results in reduced DNA-repair and diminished apoptotic rate (113, 114). As already mentioned above, ABC transporter proteins are highly expressed in liver tumors due to the missing posttranscriptional regulator miR-122 (114). All these effects caused by miR-122 downregulation promote doxorubicin resistance in liver cancer patients (113, 114). Another reason for doxorubicin resistance in liver cancer is based on reduced expression of miR-26b (161). Among the miR-26b targets in liver are the NF-κB activating proteins TAB 3 and TAK1 (161, 162). Therefore, a reduced expression of miR-26b results in increased activation of NF-κB and promotes drug resistance (161, 162). Also, downregulation of miR-101 is described as reason for resistance to doxorubicin in hepatocellular carcinoma (105). The antiapoptotic protein Mcl-1 is among the targets of miR-101 and high levels of Mcl-1 renders liver tumor cells resistant to doxorubicin treatment (105). Furthermore, doxorubicin treatment failure in liver cancer patients has been connected to downregulation of miR-199a-3p (133). Besides targeting mTOR and c-Met, miR-199a-3p influences cell cycle regulation (133). Decreased miR-199a-3p level results in downregulation of the G1-checkpoint CDK inhibitors p21 (CDKN1A) and p27 (CDKN1B) and abrogate the G1 arrest following damage to DNA (132, 133). In another study, downregulation of the G1 inhibitor CDKN1A in hepatocellular carcinoma was linked to upregulation of miR-519d (181). Consequently the apoptotic rate is reduced due to downregulated miR-199a-3p as well as upregulated miR-519d expression (133, 181).

Another important tumor suppressor protein involved in resistance to anticancer drugs is PTEN because it is a main regulator for PI3K-AKT-mTOR pathway which is often hyperactivated in cancer and is one of the drivers for tumor growth and survival (288, 289). PTEN itself is regulated by different microRNAs in different GI tumors, e.g., by miR-21 in liver and gastric cancer, miR-22 in p53-mutated colon cancer and mir-17-5p in colorectal cancer (126, 142–144, 151). In all cases, upregulation of microRNAs results in decreased PTEN level in the tumor cell and subsequent activation of AKT-mTOR pathways resulting in resistance to cisplatin (gastric cancer), paclitaxel (p53-mutated colon tumor), and FOLFOX (colorectal cancer) (126, 142–144, 151). Downregulation of PTEN due to overexpression of miR-19a and miR-19b in gastric cancer results in multi-drug resistance (134).

Furthermore, mTOR is an important regulator under physiological as well as pathological conditions. In p53 mutant colorectal cancer, mTOR is downregulated by miR-338-3p and results in resistance to 5-fluorouracil treatment (171). Indeed, inhibition of miR-338-3p in cell culture models restored sensitivity to 5-fluorouracil (171) likely due to increased autophagy and reduced apoptosis following decrease in mTOR expression (171, 290).

Autophagy is a further mechanism for chemoresistance (51, 291–293). In liver cancer, upregulation of lncRNA HULC activates autophagy by increasing the expression of ubiquitin-specific peptidase 22 (USP22) which in turn prevents the ubiquitin-mediated degradation of silent information regulator 1 (SIRT1) by removing the conjugated polyubiquitin chains from SIRT1 (51). Autophagy causes resistance to oxaliplatin, 5-fluorouracil and epitubicin treatments in liver tumors (51). In addition, lncRNA HULC downregulates the expression of microRNAs that target directly the 3′-UTR of USP22 (miR-6825-5p, miR-6845-5p, and miR-6886-3p) in liver cancer cells and prevents by this inhibition of USP22 at translational level (51).

LncRNA MALAT-1 is highly expressed in gastric cancer cells resistant to 5-fluoruracil and cis-platin, respectively, compared to parental gastric cancer cells (67). LncRNA MALAT-1 quenches miR-23b-3p and subsequently increases the expression of ATG12, an important regulator of autophagy (67).

In oxaliplatin-resistant colon cancer, miR-409-3p is downregulated so that the direct target Beclin-1 is expressed and induces autophagy (176). Overexpression of miR-409-3p results in low autophagic activity and overcomes oxaliplatin resistance in model systems of colon cancer (176).

### Induction of EMT

Drug resistance can be caused by EMT (294, 295). Several EMT-related signaling pathways are well known to be involved in mediating drug resistance in tumors (22, 295–297). Cells undergoing EMT have several features in common with cancer stem cells (e.g., increased drug efflux pumps and antiapoptotic effects) and furthermore EMT is instrumental for generation and maintenance of cancer stem cells (22, 295, 297).

The lncRNA plasmacytoma variant translocation 1 (PVT1) has been found to be elevated in nearly all GI tumors including gastric, esophageal, pancreatic, colon, and liver cancers (75–77, 298). Increased expression of lncRNA PVT1 results in EMT and drug resistance (75–77).

The tumor suppressor lncRNA LEIGC prevents normal cells to undergo EMT. Therefore, the reduced expression of lncRNA LEIGC in gastric cancer fosters EMT and results in resistance to 5-fluorouracil treatment (54, 55).

Upregulation of lncRNA HULC has been correlated to induce EMT and suppressed apoptosis in gastric tumors, leading to cisplatin resistance (52, 53).

Increased expression of lncRNA-activated by TGF-β (lncRNA-ATB) in liver cancer results in competition with members of the miR-200 family for binding sites in the 3′-UTR of mRNAs coding for the transcription factors ZEB1 and ZEB2 (33). In turn, high expression of ZEB1 and ZEB2 causes EMT and increased drug resistance (33).

In pancreatic cancer, the lncRNA MALAT-1 is a regulator of EMT (64, 65). In addition, the lncRNA MALAT-1 suppress G2/M cell cycle arrest and apoptosis leading to resistance to gemcitabine treatment (65). As demonstrated by this example, the same lncRNA can induce resistance to chemotherapy by regulating different mechanisms at the same time.

Induction of EMT and resistance to gemcitabine treatment in pancreatic cancer cells can also be caused by miR-223 overexpression (153). Inhibition of miR-223 restored the sensitivity of pancreatic cancer cell lines to gemcitabine treatment (153). Similarly, gemcitabine resistance in pancreatic cancer can also be caused by downregulation of microRNAs as demonstrated for miR-200 (miR-200a, miR-200b, and miR-200c) and let-7 family resulting in EMT (100, 135).

In colon cancer cells, downregulation of miR-147 results in EMT and increases the phosphorylation rate of AKT (123). Beside the activation of the PI3K-AKT pathway, the lower expression level of miR-147 also activates the TGF-β pathway and eventually leads to resistance to gefitinib treatment (123). Increased expression of miR-224 in colon cancer tissue was identified as another reason for resistance to 5-fluorouracil treatment. Increased miR-224 expression translates in increasing phosphorylation rate of ERK and AKT, resulting in activation of both pathways (155). In addition, miR-224 seems to activate also EGFR dependent- and NF-κB-signaling pathway leading to EMT (155).

### Cancer Cell Stemness

A further reason for drug resistance is the presence of cancer stem cells. Cancer stem cells are well known for being refractory to chemotherapies and therefore cause therapy failure and tumor recurrence or progression (299–305). Once again non-coding RNAs especially lncRNAs and microRNAs are involved in sustaining the cancer stem cell niche (95, 306–309).

The lncRNA urothelial carcinoma associated 1 [identical with lncRNA CUDR (cancer upregulated drug resistant)] is strongly expressed in different tumors; among these, gastric, hepatocellular, pancreatic, colorectal cancers, and esophageal squamous cell carcinoma (94–96, 310–314). LncRNA UCA1 binds to several microRNAs in different tumors (e.g., miR-216b in liver cancer, miR-204 in esophageal and colon cancer, miR-27b in gastric cancer) and influences entire transcriptional programs as well as response toward therapy (90, 92, 312, 314, 315). Well-established upregulated targets of lncRNA UCA1 are members of the Wnt-β-catenin signaling pathway, several transcription factors and cell division regulators (87, 93). For stem cells, the Wnt-β-catenin pathway is of pivotal importance for cell self-renewal and mediating drug resistance (316, 317). Overexpression of lncRNA UCA1 results in resistance to cancer treatments with tamoxifen, 5-fluorouracil, gemcitabine, cisplatinum, doxorubicin, imatinib, and tyrosine-kinase inhibitors targeting EGFR (90, 94, 96, 314).

Silencing of lncRNA UCA1 in *in vitro* and *in vivo* systems proved the oncogenic role of lncRNA UCA1 in gastric cancer (94, 96). Reduced expression level of lncRNA UCA1 results in reduced proliferation rate, increased apoptosis rate and overcomes the resistance to doxorubicin (94, 96). Furthermore, lncRNA UCA1 is a direct regulator of the PI3K-AKT-mTOR pathway (96) which is often found to be deregulated in human cancers and is known to contribute to chemoresistance of cancer cells (318, 319). In another study, overexpression of lncRNA UCA1 was shown to cause reduced miR-27 expression causing diminished apoptosis of gastric cancer cells due to increased Bcl-2 protein level in combination with reduced cleaved caspase-3 (92). This results in multidrug resistance of gastric tumors (92).

Overexpression of lncRNA UCA1 is also a reason for chemoresistance against 5-fluorouracil treatment in colon cancer (90). LncRNA UCA1 causes resistance by binding miR-204-5p and consequently upregulating the expression of its target genes Bcl-2, RAB22A, and CREB1 (90). miR-21 was identified as an important player in regard to failure of 5-fluorouracil therapy in colon cancer patients (139). miR-21 is able to increase the number of undifferentiated cancer stem cells during 5-fluorouracil treatment and contributes by this to therapy failure (139).

In liver cancer, lncRNA UCA1 contributes to chemotherapy resistance and malignant transformation of hepatocyte-stem cells (88, 93, 95, 320–322). LncRNA UCA1 increases directly the transcription rate of the oncogene c-myc well known to be involved in drug resistance as well as in activating stem-cell like properties in hepatocarcinoma (86, 89, 323–325). Furthermore, lncRNA UCA1 also induces the expression of lncRNA HULC (highly upregulated in liver cancer) in liver cancer and lncRNA HULC in turn stimulates the activity of the Wnt-β-catenin pathway (88). In addition, lncRNA UCA1 forms a complex with the cell-cycle regulator cyclin-D which enhances the expression of lncRNA H19 by inhibiting the methylation of the lncRNA H19 promoter (89, 95). High level of lncRNA H19 induces the telomerase activity and enhances the length of telomere thereby supporting the stem cell properties (35, 89, 326). Another effect of lncRNA UCA1 is the enhanced phosphorylation of the tumor suppressor retinoblastoma protein 1 (RB1). RB1 phosphorylation results in increased cell cycle progression and in interaction of the phosphorylated retinoblastoma protein 1 with the SET1A complex. Such interaction catalyses the transcription-activating methylation of histone H3 lysine-4 on several gene promoters including telomeric repeat-binding factor 2 promoter an important component for the telomerase extension process (91, 320).

In liver cancer as well as in pancreatic, gastric, esophageal, and colon cancers a critical role in inducing the transformation of stem cells into cancer stem cell has been demonstrated for lncRNA HOTAIR (45, 95, 327–331). LncRNA HOTAIR is a strong activator for expression of *OCT4, RNF51, CD44*, and *CD133* genes—all these proteins are involved in reprogramming the gene network to acquire cancer stem cell properties (46, 47). LncRNA HOTAIR expression causes resistance against cisplatin and doxorubicin treatment in liver cancer model systems (332) and renders gastric tumors resistant to cisplatin therapy by binding miR-126 and activating the PI3K-AKT-mTOR pathway (48). In the context of several GI cancer stem cells, it has been shown that lncRNA HOTAIR downregulates the expression of histone methyltransferase SETD2 and reduces the phosphorylation rate of SETD2 resulting in reduced trimethylation of histone H3 lysine-36 on several gene promoter, e.g., Wnt inhibitory factor-1 (WIF-1) (44, 45, 331, 333). Reduced WIF-1 expression leads to activation and increased signaling through the Wnt-β-catenin pathway (44, 45). Furthermore, the modulated chromatin organization account for a reduced efficiency of the mismatch repair system and damaged DNA can escape from corrections leading to microsatellite instability (MSI) and altered expression of cell cycle regulators as well as reduced apoptosis (124, 327, 331, 334, 335). In addition, lncRNA HOTAIR induces accumulation of replication errors by hindering the complex formation of MSH2 with MSH6; one essential dimer for DNA mismatch recognition and repair (42, 43, 124, 138, 336).

In pancreatic cancer, the oncogenic lncRNA MALAT-1 contributes to the expression of the cancer stem cell marker CD133, CD44, CD24, and aldehyde-dehydrogenase (65, 66, 337). In addition, the expression of the core pluripotent factors OCT4, NANOG, and SOX2 are also under the control of lncRNA MALAT-1 (66). LncRNA long intergenic ncRNA regulator of reprogramming (linc-ROR) inhibits the expression of p53 and activates by this the transcription factor ZEB1 in pancreatic cancer (56). ZEB1 in turn suppress the expression of the miR-200 family that leads to maintenance of pancreatic cancer stemness and induces EMT known to be responsible for paclitaxel resistance in pancreatic cancer patients (56, 57). Downregulation of miR-205 results in increased expression of stem cell markers OKT3, OKT8, and CD44 in pancreatic cancer tissue and is linked to gemcitabine resistance (137). Re-expression of miR-205 is able to overcome the gemcitabine resistance in pancreatic cancer model systems (137).

The lncRNA-34a mediates an increase in self-renewal of colon cancer stem cells and induce Wnt as well as NOTCH signaling pathways *via* sequester miR-34a expression (98, 99).

In hepatocellular carcinoma, the lncRNA is involved in regulating core pluripotent factors (OCT-4, NANOG, SOX2) necessary for the stem cell like phenotype and causes resistance to chemotherapy (59). LncRNA linc-ROR competes with miR-145 for the same binding sites present in the mRNAs coding for OCT-4, NANOG, and SOX2 (58). Presence of lncRNA linc-ROR prevents the binding of miR-145 to the mRNA of the core pluripotent factors resulting in translation of these mRNAs and maintains the stem cell phenotype (58). Furthermore, the expression of CD133, another cancer stem cell marker, is directly induced by lncRNA linc-ROR (59).

miR-130b is connected to cancer stem cells growth in liver tumors (119). Increased expression of miR-130b targets directly the mRNA coding for tumor protein 53-induced nuclear protein 1 and reduces the expression level of the corresponding protein (119). Furthermore, high level of miR-130b renders liver tumor cells resistant to doxorubicin treatment (119). Another reason for doxorubicin resistance in liver cancer patients is downregulation of the tumor suppressor miR-101 resulting in increased protein expression of enhancer of zeste homolog 2 (EZH2) (103, 104). EZH2 is a histone-lysine N-methyltransferase enzyme that silence Wnt-pathway antagonists and other tumor suppressor genes on the transcriptional level by histone methylation (338). Overexpression of EZH2 is positively correlated with increased Wnt-β-catenin signaling (338).

miR-221 is over-expressed in 5-fluorouracil-resistant esophageal tumors (152). The mechanisms of resistance is mediated *via* downregulation of the direct target dickkopf-related protein 2 (DDK2) and subsequent activation of the Wnt-β-catenin pathway (152). Furthermore, increased miR-221 expression fosters EMT and facilitates the formation of tumor stem cells (152).

In colon cancer stem cells, miR-451 was found to be downregulated compared to colon cancer cells (177). Reduced level of miR-451 seems to be essential for the self-renewal of colon cancer stem cells (177). In addition, expression of ABCB1 transporter is increased in colon cancer stem cells due to lack of miR-451 posttranscriptional downregulation resulting in resistance to irinotecan treatment (177).

miR-1182 is often downregulated in gastric cancer tissue (112). One direct target of miR-1182 is telomerase reverse transcriptase (hTERT), an enzyme that is involved in controlling the length of telomere. Overexpression of hTERT due to missing transcriptional regulation by miR-1182, results in cell immortality and stem-cell property of gastric cancer cells (112).

### Targeted Therapies and Drug Resistance

For GI cancer several targeted therapies exist (Table 3) (339–345). They are used alone or in combination with chemotherapy. Unfortunately in most cases the patients develop resistance also against these targeted therapies and the above outlined general principles of drug resistance based on non-coding RNA dysregulation are involved. Beside that non-coding RNAs interfering with the targeted protein itself or (up-)regulating the targeted signal pathway are involved in drug resistance (342). Furthermore, therapy failure can be related to activation of alternative signal pathways by non-coding RNAs (68, 342).

**Table 3:** Approved targeted therapies for GI cancer.

GI cancer	Drug	Target
Gastric cancer	Trastuzumab	HER2
	Ramucirumab	VEGFR-2
	Pembrolizumab	PD-1
Hepatocellular cancer	Sorafenib	RAF, VEGFR-2, VEGFR-3, PDGFR, c-KIT
Colon cancer	Cetuximab, panitumumab	EGFR
	Bevacizumab	VEGF
	Regorafenib	VEGFR-1, VEGFR-2, VEGFR-3, BRAF, c-KIT, RET, PDGFR
Colon cancer with MSI-H	Pembrolizumab	PD-1

*HER2, human epidermal growth factor receptor 2; VEGFR, vascular endothelial growth factor receptor; PD-1, programmed cell death protein-1; RAF, rapidly accelerated fibrosarcoma; PDGFR, platelet-derived growth factor receptor; c-KIT, SCFR, mast/stem cell growth factor receptor; EGFR, epidermal growth factor receptor; VEGF, vascular endothelial growth factor; RET, rearranged during transfection; MSI-H, microsatellite instability-high*.

Recently, it was demonstrated that resistance to cetuximab in colon cancer patients and in *in vitro* 3-D-cell culture models can be caused by overexpression of lncRNA MIR100HG (68). Two microRNAs, miR-100, and miR-125b, are generated from lncRNA MIR100HG and these microRNAs downregulate in a concerted way five negative regulators of the Wnt/β-catenin pathway resulting in increased Wnt signaling (68). This kind of cetuximab resistance can be overcome by inhibition of Wnt signaling, underscoring the potential clinical relevance of the interactions between EGFR and Wnt/β-catenin pathways (68). Increased mir-125b expression is also correlated with trastuzumab resistance in HER2-positive gastric cancer patients but up to now the molecular basis for this resistance is unclear (346). Sorafenib resistance in hepatocellular carcinoma is caused by lncRNA TUC338 (78). RAS protein activator like-1 (RASAL-1) is a direct target of lncRNA TUC338 and high expression of lncRNA TUC338 inhibits the RASAL-1 expression resulting in activation of RAS-signaling (78). According to another *in vitro* study, reduced expression of miR-193b leads to higher expression of the antiapoptotic protein Mcl-1 and renders hepatocellular carcinoma cells resistant to sorafenib treatment (130).

### Non-Coding RNAs as Potential Biomarkers of Resistance and Novel Therapeutics: Promises and Hurdles

Our review summarizes most of the current evidence supporting the role of non-coding RNAs in resistance to chemotherapy and targeted agents. It is likely that, in the near future, given the promising and exciting results obtained with the use of immunotherapy in gastroesophageal (347) and colorectal cancer (348, 349), new data will emerge on the already known regulation of PD-1, PD-L1, and CTLA-4 by non-coding RNAs and response to nivolumab and pembrolizumab (350–352).

The contribution of non-cording RNAs in resistance mechanisms to a broad range of anticancer treatments makes their use as biomarkers or novel therapeutics quite promising but several challenges remain.

Given microRNAs and, to a lesser extent, other non-coding RNAs can be reliably detected in tissues and biofluids such as plasma, serum, and urine, it is tempting to hypothesize the use of non-coding RNA based tools to predict and monitor resistance to anticancer treatments. Few studies have already tested the validity of microRNAs as biomarkers of response to anticancer treatment in other cancers such as prostate (353), chronic lymphocytic leukemia (354), and sarcomas (355). In colorectal cancer, we (356) and others (357–359) have tested the contribution of a single nucleotide polymorphism (SNP) in the binding site of let-7 in the *KRAS* 3’UTR in predicting benefit from anti-EGFR treatment with conflicting results across different trials. Despite the good reproducibility of the assay, the predictive value of the test was not confirmed in all trials likely due to use of cetuximab in different context (neoadjuvant, adjuvant and metastatic colorectal cancer, respectively). Similarly the analysis of a SNP in miR-608 led to contradicting results in patients treated with neoadjuvant or adjuvant chemo- and radiochemotherapy in colon and rectal cancers highlighting some of the challenges in validating data obtained in retrospective series (360–363). Tissue (cancer *versus* stroma) and organ (colon *versus* rectum) specificity in non-coding RNA expression might represent potential explanations for different findings obtained in some of these studies. Beside SNPs, expression of microRNAs can be detected in fresh frozen or formalin fixed paraffin embedded tissues and serve as potential biomarker of sensitivity or resistance to treatment. Robust data have emerged from the retrospective analysis of a prospective phase III clinical trial (364). In this study, *KRAS* wild-type patients were classified based on high or low miR-31-3p expression: patients with high expression were resistant to cetuximab while patient with low expression had good and durable responses which translated in survival benefit. The miR-31 expression cutoff for the classification into high or low expression was predefined in the above study. However, one of the key challenges in validating these interesting findings will be design of a clinically approved assay that can accurately assign patients into one of these two categories. In this prospective, the use of different sources of material (i.e., primary colorectal cancer *versus* metastasis) might result in different basal expression of the microRNA and as such different scoring. Source of material and choice of reference controls represent important obstacles that might bias the definition of a threshold for high or low expression of microRNAs in tissues and biofluids. MicroRNAs can be detected in plasma, serum and urine samples and have been used for early detection and prognostic purposes in GI cancer (365–367). The use of digital droplet approaches allows the quantitative detection of copies of the microRNA of interest based on the starting volume of biofluids and, potentially overcomes or at least mitigates, the issues related to the normalization of data against reference controls, making the definition of cutoff easier to standardize. One study has reported the potential role of miR-126 in predicting and tracking response to chemotherapy and anti-VEGF treatment in colorectal cancer (368) and, with the advent of digital quantitative technologies, more studies are expected.

In consideration of their role in cancer initiation, progression and resistance to treatment, non-coding RNAs and among them microRNAs have been proposed as potential therapeutics (369). A large body of pre-clinical evidence is available on the use of anti-microRNAs or molecules re-expressing microRNAs alone or in combination with other agents in order to increase efficacy and prevent or revert drug resistance (370). Inhibition of microRNAs has been tested in clinical trials in the context of HCV infection (371, 372) and in mesothelioma (373). These trials highlighted a huge potential for microRNA-based therapeutics but at the same time pinpointed some of the criticalities in further clinical development of such approaches. miR-122 inhibition led to durable viral load reduction in both HCV trials and was associated with manageable side effects. Similarly, in mesothelioma patients treated with miR-16-loaded minicells the disease control rate was satisfactory and the toxicity profile acceptable warranting further investigations. Overall in both approaches the risk of off-target effects represent the main hurdle to be taken into account: indeed miR-122 inhibition has been associated with risk of developing liver cancer in preclinical models (374) and, similarly, overexpression of miR-16 might lead to uncontrolled cardiac effects as proven in the phase I trial (373). These effects might be increased in combination studies in which anti-microRNAs or microRNA-conjugates are delivered together with chemotherapy leading to cumulative side effects. Therefore, a robust understanding of the biology underpinning microRNA deregulation in physiology and pathological conditions in order to implement effort that can minimize the risk of serious adverse events hampering the clinical development of microRNA-based strategies.

## Conclusion

Non-coding RNAs especially lncRNAs and microRNAs are important mediators for drug resistance. They function in an organ and tissue specific manner and through different molecular mechanisms. One non-coding RNA always have several targets and in the end deregulation of one non-coding RNA alters the expression level of several proteins in a tissue specific way. For example, in the case of miR-374b more than 700 genes have been identified as direct target in pancreatic tissue (174). Drug resistance is a dynamic process caused by several cell and non-cell autonomous mechanisms. Given non-coding RNAs can simultaneously control several cancer-associated pathways, non-coding RNA dysregulation plays a crucial role in treatment resistance. Future studies will continue to shed insights in the fine interplay among lncRNA, microRNA and their target genes and might provide opportunities for more effective strategies to prevent or overcome resistance. In the interim, given non-coding RNAs and especially microRNAs can be tested in tissues and biofluids in a rapid, cost/effective and robust way. More investigational studies should explore their utility to monitor and forecast treatment response and resistance in order to personalize treatments and improve patient’s outcomes.

## Author Contributions

NV and JCH: idea, conception, and writing the review.

## Conflict of Interest Statement

The authors declare that the research was conducted in the absence of any commercial or financial relationships that could be construed as a potential conflict of interest.

## References

[1] Pourhoseingholi MA Vahedi M Baghestani AR. Burden of gastrointestinal cancer in Asia; an overview. Gastroenterol Hepatol Bed Bench (2015) 8:19–27. 25584172 PMC4285928

[2] Siegel RL Miller KD Jemal A. Cancer statistics, 2015. CA Cancer J Clin (2015) 65:5–29. 10.3322/caac.21254 25559415

[3] Torre LA Bray F Siegel RL Ferlay J Lortet-Tieulent J Jemal A. Global cancer statistics, 2012. CA Cancer J Clin (2015) 65:87–108. 10.3322/caac.21262 25651787

[4] Hung AY Canning CA Patel KM Holland JM Kachnic LA. Radiation therapy for gastrointestinal cancer. Hematol Oncol Clin North Am (2006) 20:287–320. 10.1016/j.hoc.2006.01.016 16730296

[5] Chan BA Jang RW Wong RK Swallow CJ Darling GE Elimova E. Improving outcomes in resectable gastric cancer: a review of current and future strategies. Oncology (Williston Park) (2016) 30:635–45. 27422110

[6] Ismael HN Denbo J Cox S Crane CH Das P Krishnan S Biologic mesh spacer placement facilitates safe delivery of dose-intense radiation therapy: a novel treatment option for unresectable liver tumors. Eur J Surg Oncol (2016) 42:1591–6. 10.1016/j.ejso.2016.05.021 27296729

[7] Jakhetiya A Garg PK Prakash G Sharma J Pandey R Pandey D. Targeted therapy of gastrointestinal stromal tumours. World J Gastrointest Surg (2016) 8:345–52. 10.4240/wjgs.v8.i5.345 27231512 PMC4872062

[8] Murphy MB Adjunctive therapy of gastric cancer: moving the field forward. Oncology (Williston Park) (2016) 30:646–7. 27422111

[9] Olcina MM Giaccia AJ. Reducing radiation-induced gastrointestinal toxicity – the role of the PHD/HIF axis. J Clin Invest (2016) 126:3708–15. 10.1172/JCI84432 27548524 PMC5096800

[10] Rautio T Kairaluoma M Sand J [Novel techniques in the treatment of rectal cancer]. Duodecim (2016) 132:1160–4. 27483632

[11] Ristamaki R Algars A. [Principles of oncologic drug therapy following surgery for bowel cancer]. Duodecim (2016) 132:1155–9. 27483631

[12] Rutkowski P Hompes D. Combined therapy of gastrointestinal stromal tumors. Surg Oncol Clin N Am (2016) 25:735–59. 10.1016/j.soc.2016.05.006 27591496

[13] Slamon DJ Leyland-Jones B Shak S Fuchs H Paton V Bajamonde A Use of chemotherapy plus a monoclonal antibody against HER2 for metastatic breast cancer that overexpresses HER2. N Engl J Med (2001) 344:783–92. 10.1056/NEJM200103153441101 11248153

[14] Motzer RJ Hutson TE Tomczak P Michaelson MD Bukowski RM Rixe O Sunitinib versus interferon alfa in metastatic renal-cell carcinoma.N Engl J Med (2007) 356:115–24. 10.1056/NEJMoa065044 17215529

[15] Blanke CD Demetri GD Von Mehren M Heinrich MC Eisenberg B Fletcher JA Long-term results from a randomized phase II trial of standard- versus higher-dose imatinib mesylate for patients with unresectable or metastatic gastrointestinal stromal tumors expressing KIT. J Clin Oncol (2008) 26:620–5. 10.1200/JCO.2007.13.4403 18235121

[16] Maemondo M Inoue A Kobayashi K Sugawara S Oizumi S Isobe H Gefitinib or chemotherapy for non-small-cell lung cancer with mutated EGFR. N Engl J Med (2010) 362:2380–8. 10.1056/NEJMoa0909530 20573926

[17] Chapman PB Hauschild A Robert C Haanen JB Ascierto P Larkin J Improved survival with vemurafenib in melanoma with BRAF V600E mutation. N Engl J Med (2011) 364:2507–16. 10.1056/NEJMoa1103782 21639808 PMC3549296

[18] Kwak EL Bang YJ Camidge DR Shaw AT Solomon B Maki RG Anaplastic lymphoma kinase inhibition in non-small-cell lung cancer. N Engl J Med (2010) 363:1693–703. 10.1056/NEJMoa1006448 20979469 PMC3014291

[19] Douillard JY Rong A Sidhu R RAS mutations in colorectal cancer. N Engl J Med (2013) 369:2159–60. 10.1056/NEJMoa1305275 24283232

[20] Korpanty GJ Graham DM Vincent MD Leighl NB. Biomarkers that currently affect clinical practice in lung cancer: EGFR, ALK, MET, ROS-1, and KRAS. Front Oncol (2014) 4:204. 10.3389/fonc.2014.00204 25157335 PMC4127527

[21] Siroy AE Boland GM Milton DR Roszik J Frankian S Malke J Beyond BRAF(V600): clinical mutation panel testing by next-generation sequencing in advanced melanoma. J Invest Dermatol (2015) 135:508–15. 10.1038/jid.2014.366 25148578 PMC4289407

[22] Housman G Byler S Heerboth S Lapinska K Longacre M Snyder N Drug resistance in cancer: an overview. Cancers (Basel) (2014) 6:1769–92. 10.3390/cancers6031769 25198391 PMC4190567

[23] Zahreddine H Borden KL Mechanisms and insights into drug resistance in cancer. Front Pharmacol (2013) 4:28 10.3389/fphar.2013.00028 23504227 PMC3596793

[24] Shang Y Cai X Fan D. Roles of epithelial-mesenchymal transition in cancer drug resistance. Curr Cancer Drug Targets (2013) 13:915–29. 10.2174/15680096113136660097 24168191

[25] Xia H Hui KM. Mechanism of cancer drug resistance and the involvement of noncoding RNAs. Curr Med Chem (2014) 21:3029–41. 10.2174/0929867321666140414101939 24735364

[26] Mitra A Mishra L Li S. EMT, CTCs and CSCs in tumor relapse and drug-resistance. Oncotarget (2015) 6:10697–711. 10.18632/oncotarget.4037 25986923 PMC4484413

[27] Prieto-Vila M Takahashi RU Usuba W Kohama I Ochiya T. Drug resistance driven by cancer stem cells and their niche. Int J Mol Sci (2017) 18:2574–96. 10.3390/ijms18122574 29194401 PMC5751177

[28] Hang Q Sun R Jiang C Li Y. Notch 1 promotes cisplatin-resistant gastric cancer formation by upregulating lncRNA AK022798 expression. Anticancer Drugs (2015) 26:632–40. 10.1097/CAD.0000000000000227 25763542

[29] Zhang XW Bu P Liu L Zhang XZ Li J Overexpression of long non-coding RNA PVT1 in gastric cancer cells promotes the development of multidrug resistance. Biochem Biophys Res Commun (2015) 462:227–32. 10.1016/j.bbrc.2015.04.121 25956062

[30] Lan WG Xu DH Xu C Ding CL Ning FL Zhou YL Silencing of long non-coding RNA ANRIL inhibits the development of multidrug resistance in gastric cancer cells. Oncol Rep (2016) 36:263–70. 10.3892/or.2016.4771 27121324

[31] Jiang M Huang O Xie Z Wu S Zhang X Shen A A novel long non-coding RNA-ARA: adriamycin resistance-associated. Biochem Pharmacol (2014) 87:254–83. 10.1016/j.bcp.2013.10.020 24184505

[32] Cox J Weinman S. Mechanisms of doxorubicin resistance in hepatocellular carcinoma. Hepat Oncol (2016) 3:57–9. 10.2217/hep.15.41 26998221 PMC4792121

[33] Yuan JH Yang F Wang F Ma JZ Guo YJ Tao QF A long noncoding RNA activated by TGF-beta promotes the invasion-metastasis cascade in hepatocellular carcinoma. Cancer Cell (2014) 25:666–81. 10.1016/j.ccr.2014.03.010 24768205

[34] Ma YL Yang YZ Wang F Moyer MP Wei Q Zhang P Long non-coding RNA CCAL regulates colorectal cancer progression by activating Wnt/beta-catenin signalling pathway via suppression of activator protein 2 alpha. Gut (2016) 65:1494–504. 10.1136/gutjnl-2014-308392 25994219

[35] Hiyama E Hiyama K. Telomere and telomerase in stem cells. Br J Cancer (2007) 96:1020–4. 10.1038/sj.bjc.6603671 17353922 PMC2360127

[36] Tsang WP Kwok TT. Riboregulator H19 induction of MDR1-associated drug resistance in human hepatocellular carcinoma cells. Oncogene (2007) 26:4877–81. 10.1038/sj.onc.1210266 17297456

[37] Matouk I Raveh E Ohana P Abu Lail R Gershtain E Gilon M The increasing complexity of the oncofetal H19 gene locus: functional dissection and therapeutic intervention. Int J Mol Sci (2013) 14:4298–316. 10.3390/ijms14024298 23429271 PMC3588099

[38] El-Deiry WS Tokino T Velculescu VE Levy DB Parsons R Trent JM WAF1, a potential mediator of p53 tumor suppression. Cell (1993) 75:817–25. 10.1016/0092-8674(93)90500-P 8242752

[39] Geng YJ Xie SL Li Q Ma J Wang GY Large intervening non-coding RNA HOTAIR is associated with hepatocellular carcinoma progression. J Int Med Res (2011) 39:2119–28. 10.1177/147323001103900608 22289527

[40] Kogo R Shimamura T Mimori K Kawahara K Imoto S Sudo T Long noncoding RNA HOTAIR regulates polycomb-dependent chromatin modification and is associated with poor prognosis in colorectal cancers. Cancer Res (2011) 71:6320–6. 10.1158/0008-5472.CAN-11-1021 21862635

[41] Liu ZL Sun M Lu KH Liu J Zhang ML Wu WQ The long noncoding RNA HOTAIR contributes to cisplatin resistance of human lung adenocarcinoma cells via downregualtion of p21(WAF1/CIP1) expression. PLoS One (2013) 8:e77293. 10.1371/journal.pone.0077293 24155936 PMC3796503

[42] Yang Q Zhang R Wang XW Linke SP Sengupta S Hickson ID The mismatch DNA repair heterodimer, hMSH2/6, regulates BLM helicase. Oncogene (2004) 23:3749–56. 10.1038/sj.onc.1207462 15064730

[43] Edelbrock MA Kaliyaperumal S Williams KJ. Structural, molecular and cellular functions of MSH2 and MSH6 during DNA mismatch repair, damage signaling and other noncanonical activities. Mutat Res (2013) 743-744:53–66. 10.1016/j.mrfmmm.2012.12.008 23391514 PMC3659183

[44] Ge XS Ma HJ Zheng XH Ruan HL Liao XY Xue WQ HOTAIR, a prognostic factor in esophageal squamous cell carcinoma, inhibits WIF-1 expression and activates Wnt pathway. Cancer Sci (2013) 104:1675–82. 10.1111/cas.12296 24118380 PMC7653522

[45] Kim K Jutooru I Chadalapaka G Johnson G Frank J Burghardt R HOTAIR is a negative prognostic factor and exhibits pro-oncogenic activity in pancreatic cancer. Oncogene (2013) 32:1616–25. 10.1038/onc.2012.193 22614017 PMC3484248

[46] Padua Alves C Fonseca AS Muys BR De Barros ELBR Burger MC De Souza JE Brief report: the lincRNA HOTAIR is required for epithelial-to-mesenchymal transition and stemness maintenance of cancer cell lines. Stem Cells (2013) 31:2827–32. 10.1002/stem.1547 24022994

[47] Zhu Y Luo M Brooks M Clouthier SG Wicha MS. Biological and clinical significance of cancer stem cell plasticity. Clin Transl Med (2014) 3:32. 10.1186/s40169-014-0032-3 26932376 PMC4883980

[48] Yan J Dang Y Liu S Zhang Y Zhang G. LncRNA HOTAIR promotes cisplatin resistance in gastric cancer by targeting miR-126 to activate the PI3K/AKT/MRP1 genes. Tumour Biol (2016) 34:16345–55. 10.1007/s13277-016-5448-5 27900563

[49] Wang KC Yang YW Liu B Sanyal A Corces-Zimmerman R Chen Y A long noncoding RNA maintains active chromatin to coordinate homeotic gene expression. Nature (2011) 472:120–4. 10.1038/nature09819 21423168 PMC3670758

[50] Li Z Zhao X Zhou Y Liu Y Zhou Q Ye H The long non-coding RNA HOTTIP promotes progression and gemcitabine resistance by regulating HOXA13 in pancreatic cancer. J Transl Med (2015) 13:84 10.1186/s12967-015-0442-z 25889214 PMC4372045

[51] Xiong H Ni Z He J Jiang S Li X He J LncRNA HULC triggers autophagy via stabilizing Sirt1 and attenuates the chemosensitivity of HCC cells. Oncogene (2017) 36:3528–40. 10.1038/onc.2016.521 28166203

[52] Zhao Y Guo Q Chen J Hu J Wang S Sun Y Role of long non-coding RNA HULC in cell proliferation, apoptosis and tumor metastasis of gastric cancer: a clinical and in vitro investigation. Oncol Rep (2014) 31:358–64. 10.3892/or.2013.2850 24247585

[53] Zhang Y Song X Wang X Hu J Jiang L Silencing of LncRNA HULC enhances chemotherapy induced apoptosis in human gastric cancer. J Med Biochem (2016) 35:137–43. 10.1515/jomb-2015-0016 28356873 PMC5346790

[54] Han Y Ye J Wu D Wu P Chen Z Chen J LEIGC long non-coding RNA acts as a tumor suppressor in gastric carcinoma by inhibiting the epithelial-to-mesenchymal transition. BMC Cancer (2014) 14:932 10.1186/1471-2407-14-932 25496320 PMC4295322

[55] Fang XY Pan HF Leng RX Ye DQ. Long noncoding RNAs: novel insights into gastric cancer. Cancer Lett (2015) 356:357–66. 10.1016/j.canlet.2014.11.005 25444905

[56] Wellner U Schubert J Burk UC Schmalhofer O Zhu F Sonntag A The EMT-activator ZEB1 promotes tumorigenicity by repressing stemness-inhibiting microRNAs. Nat Cell Biol (2009) 11:1487–95. 10.1038/ncb1998 19935649

[57] Kim G. nab-Paclitaxel for the treatment of pancreatic cancer. Cancer Manag Res (2017) 9:85–96. 10.2147/CMAR.S127840 28356771 PMC5360414

[58] Wang Y Xu Z Jiang J Xu C Kang J Xiao L Endogenous miRNA sponge lincRNA-RoR regulates Oct4, Nanog, and Sox2 in human embryonic stem cell self-renewal. Dev Cell (2013) 25:69–80. 10.1016/j.devcel.2013.03.002 23541921

[59] Takahashi K Yan IK Kogure T Haga H Patel T. Extracellular vesicle-mediated transfer of long non-coding RNA ROR modulates chemosensitivity in human hepatocellular cancer. FEBS Open Bio (2014) 4:458–67. 10.1016/j.fob.2014.04.007 24918061 PMC4050189

[60] Tong YS Zhou XL Wang XW Wu QQ Yang TX Lv J Association of decreased expression of long non-coding RNA LOC285194 with chemoradiotherapy resistance and poor prognosis in esophageal squamous cell carcinoma. J Transl Med (2014) 12:233. 10.1186/s12967-014-0233-y 25169763 PMC4155091

[61] Smith J Tho LM Xu N Gillespie DA. The ATM-Chk2 and ATR-Chk1 pathways in DNA damage signaling and cancer. Adv Cancer Res (2010) 108:73–112. 10.1016/B978-0-12-380888-2.00003-0 21034966

[62] Lin CY Xu HM. Novel perspectives of long non-coding RNAs in esophageal carcinoma. Carcinogenesis (2015) 36:1255–62. 10.1093/carcin/bgv136 26392258

[63] Wang X Li M Wang Z Han S Tang X Ge Y Silencing of long noncoding RNA MALAT1 by miR-101 and miR-217 inhibits proliferation, migration, and invasion of esophageal squamous cell carcinoma cells. J Biol Chem (2015) 290:3925–35. 10.1074/jbc.M114.596866 25538231 PMC4326802

[64] Ying L Chen Q Wang Y Zhou Z Huang Y Qiu F. Upregulated MALAT-1 contributes to bladder cancer cell migration by inducing epithelial-to-mesenchymal transition. Mol Biosyst (2012) 8:2289–94. 10.1039/c2mb25070e 22722759

[65] Jiao F Hu H Yuan C Wang L Jiang W Jin Z Elevated expression level of long noncoding RNA MALAT-1 facilitates cell growth, migration and invasion in pancreatic cancer. Oncol Rep (2014) 32:2485–92. 10.3892/or.2014.3518 25269958

[66] Jiao F Hu H Han T Yuan C Wang L Jin Z Long noncoding RNA MALAT-1 enhances stem cell-like phenotypes in pancreatic cancer cells.Int J Mol Sci (2015) 16:6677–93. 10.3390/ijms16046677 25811929 PMC4424983

[67] Yiren H Yingcong Y Sunwu Y Keqin L Xiaochun T Senrui C Long noncoding RNA MALAT1 regulates autophagy associated chemoresistance via miR-23b-3p sequestration in gastric cancer. Mol Cancer (2017) 16:174. 10.1186/s12943-017-0743-3 29162158 PMC5699172

[68] Lu Y Zhao X Liu Q Li C Graves-Deal R Cao Z lncRNA MIR100HG-derived miR-100 and miR-125b mediate cetuximab resistance via Wnt/beta-catenin signaling. Nat Med (2017) 23:1331–41. 10.1038/nm.4424 29035371 PMC5961502

[69] Wang Y Zhang DX Wu KC Zhao QC Nie YZ Fan DM. Long noncoding RNA MRUL promotes ABCB1 expression in multidrug-resistant gastric cancer cell sublines. Mol Cell Biol (2014) 34:3182–93. 10.1128/MCB.01580-13 24958102 PMC4135559

[70] Hung T Wang YL Lin MF Koegel AK Kotake Y Grant GD Extensive and coordinated transcription of noncoding RNAs within cell-cycle promoters. Nat Genet (2011) 43:621–9. 10.1038/ng.848 21642992 PMC3652667

[71] Peng W Fan H. Long non-coding RNA PANDAR correlates with poor prognosis and promotes tumorigenesis in hepatocellular carcinoma. Biomed Pharmacother (2015) 72:113–8. 10.1016/j.biopha.2015.04.014 26054684

[72] Ma P Xu TP Huang MD Shu YQ Increased expression of LncRNA PANDAR predicts a poor prognosis in gastric cancer. Biomed Pharmacother (2016) 78:172–6. 10.1016/j.biopha.2016.01.025 26898439

[73] Lu M Liu Z Li B Wang G Li DC Zhu YP The high expression of long non-coding RNA PANDAR indicates a poor prognosis for colorectal cancer and promotes metastasis by EMT pathway. J Cancer Res Clin Oncol (2017) 143:71–81. 10.1007/s00432-016-2252-y 27629879 PMC11818975

[74] Xu Y Jiang XM Cui YF Upregulated long noncoding RNA PANDAR predicts an unfavorable prognosis and promotes tumorigenesis in cholangiocarcinoma. Onco Targets Ther (2017) 10:2873–83. 10.2147/OTT.S137044 28652769 PMC5476724

[75] Zheng X Hu H Li S. High expression of lncRNA PVT1 promotes invasion by inducing epithelial-to-mesenchymal transition in esophageal cancer. Oncol Lett (2016) 12:2357–62. 10.3892/ol.2016.5026 27698800 PMC5038502

[76] Wu BQ Jiang Y Zhu F Sun DL He XZ Long noncoding RNA PVT1 promotes EMT and cell proliferation and migration through downregulating p21 in pancreatic cancer cells. Technol Cancer Res Treat (2017) 16:819–27. 10.1177/1533034617700559 PMC5762037 28355965

[77] Zhou DD Liu XF Lu CW Pant OP Liu XD. Long non-coding RNA PVT1: emerging biomarker in digestive system cancer. Cell Prolif (2017) 50:e12398–405. 10.1111/cpr.12398 29027279 PMC6529066

[78] Jin W Chen L Cai X Zhang Y Zhang J Ma D Long non-coding RNA TUC338 is functionally involved in sorafenib-sensitized hepatocarcinoma cells by targeting RASAL1. Oncol Rep (2017) 37:273–80. 10.3892/or.2016.5248 27878301

[79] Huang MD Chen WM Qi FZ Sun M Xu TP Ma P Long non-coding RNA TUG1 is up-regulated in hepatocellular carcinoma and promotes cell growth and apoptosis by epigenetically silencing of KLF2. Mol Cancer (2015) 14:165. 10.1186/s12943-015-0431-0 26336870 PMC4558931

[80] Dong R Liu GB Liu BH Chen G Li K Zheng S Targeting long non-coding RNA-TUG1 inhibits tumor growth and angiogenesis in hepatoblastoma. Cell Death Dis (2016) 7:e2278. 10.1038/cddis.2016.143 27362796 PMC5108331

[81] Jiang L Wang WC Li GL Sun CL Ren ZQ Sheng HH High TUG1 expression is associated with chemotherapy resistance and poor prognosis in esophageal squamous cell carcinoma. Cancer Chemother Pharmacol (2016) 78:333–9. 10.1007/s00280-016-3066-y 27329359

[82] Li Z Shen JX Chan MTV Wu WKK TUG1: a pivotal oncogenic long non-coding RNA of human cancers. Cell Prolif (2016) 49:471–5. 10.1111/cpr.12269 27339553 PMC6496395

[83] Wang L Zhao ZX Feng WD Ye ZJ Dai WG Zhang CH Long non-coding RNA TUG1 promotes colorectal cancer metastasis via EMT pathway. Oncotarget (2016) 7:51713–9. 10.18632/oncotarget.10563 27421138 PMC5239509

[84] Zhang E He X Yin D Han L Qiu M Xu T Increased expression of long noncoding RNA TUG1 predicts a poor prognosis of gastric cancer and regulates cell proliferation by epigenetically silencing of p57. Cell Death Dis (2016) 7:e2109–19. 10.1038/cddis.2015.356 PMC4849144 26913601

[85] Xu Y Leng KM Li ZL Zhang FM Zhong XY Kang PC The prognostic potential and carcinogenesis of long non-coding RNA TUG1 in human cholangiocarcinoma. Oncotarget (2017) 8:65823–35. 10.18632/oncotarget.19502 29029475 PMC5630375

[86] Walker TL White JD Esdale WJ Burton MA Decruz EE. Tumour cells surviving in vivo cisplatin chemotherapy display elevated c-myc expression. Br J Cancer (1996) 73:610–4. 10.1038/bjc.1996.105 8605094 PMC2074343

[87] Wang F Li X Xie X Zhao L Chen W. UCA1, a non-protein-coding RNA up-regulated in bladder carcinoma and embryo, influencing cell growth and promoting invasion. FEBS Lett (2008) 582:1919–27. 10.1016/j.febslet.2008.05.012 18501714

[88] Gui X Li H Li T Pu H Lu D Long noncoding RNA CUDR regulates HULC and beta-catenin to govern human liver stem cell malignant differentiation. Mol Ther (2015) 23:1843–53. 10.1038/mt.2015.166 26347501 PMC4700108

[89] Pu H Zheng Q Li H Wu M An J Gui X CUDR promotes liver cancer stem cell growth through upregulating TERT and C-Myc. Oncotarget (2015) 6:40775–98. 10.18632/oncotarget.5805 26513297 PMC4747368

[90] Bian Z Jin L Zhang J Yin Y Quan C Hu Y LncRNA-UCA1 enhances cell proliferation and 5-fluorouracil resistance in colorectal cancer by inhibiting miR-204-5p. Sci Rep (2016) 6:23892. 10.1038/srep23892 27046651 PMC4820696

[91] Fang L Zhang J Zhang H Yang X Jin X Zhang L H3K4 methyltransferase set1a is a key Oct4 coactivator essential for generation of Oct4 positive inner cell mass. Stem Cells (2016) 34:565–80. 10.1002/stem.2250 26785054

[92] Fang Q Chen X Zhi X Long non-coding RNA (LncRNA) urothelial carcinoma associated 1 (UCA1) increases multi-drug resistance of gastric cancer via downregulating miR-27b. Med Sci Monit (2016) 22:3506–13. 10.12659/MSM.900688 27694794 PMC5051552

[93] Li CH Chen Y. Insight into the role of long noncoding RNA in cancer development and progression. Int Rev Cell Mol Biol (2016) 326:33–65. 10.1016/bs.ircmb.2016.04.001 27572126

[94] Shang C Guo Y Zhang J Huang B. Silence of long noncoding RNA UCA1 inhibits malignant proliferation and chemotherapy resistance to adriamycin in gastric cancer. Cancer Chemother Pharmacol (2016) 77:1061–7. 10.1007/s00280-016-3029-3 27056384

[95] Chen S Zhu J Wang F Guan Z Ge Y Yang X LncRNAs and their role in cancer stem cells. Oncotarget (2017) 8:110685–92. 10.18632/oncotarget.22161 29299179 PMC5746414

[96] Li C Liang G Yang S Sui J Yao W Shen X Dysregulated lncRNA-UCA1 contributes to the progression of gastric cancer through regulation of the PI3K-Akt-mTOR signaling pathway. Oncotarget (2017) 8:93476–91. 10.18632/oncotarget.19281 29212166 PMC5706812

[97] Xu WH Zhang JB Dang Z Li X Zhou T Liu J Long non-coding RNA URHC regulates cell proliferation and apoptosis via ZAK through the ERK/MAPK signaling pathway in hepatocellular carcinoma. Int J Biol Sci (2014) 10:664–76. 10.7150/ijbs.8232 25013376 PMC4081602

[98] Bu P Chen KY Chen JH Wang L Walters J Shin YJ A microRNA miR-34a-regulated bimodal switch targets Notch in colon cancer stem cells. Cell Stem Cell (2013) 12:602–15. 10.1016/j.stem.2013.03.002 23642368 PMC3646336

[99] Evans J Essex A Xin H Amitai N Brinton L Griner E Registered report: Wnt activity defines colon cancer stem cells and is regulated by the microenvironment. Elife (2015) 4:e07301–6. 10.7554/eLife.07301 26287525 PMC4541490

[100] Li Y Vandenboom TG II Kong D Wang Z Ali S Philip PA Up-regulation of miR-200 and let-7 by natural agents leads to the reversal of epithelial-to-mesenchymal transition in gemcitabine-resistant pancreatic cancer cells. Cancer Res (2009) 69:6704–12. 10.1158/0008-5472.CAN-09-1298 19654291 PMC2727571

[101] Bhutia YD Hung SW Krentz M Patel D Lovin D Manoharan R Differential processing of let-7a precursors influences RRM2 expression and chemosensitivity in pancreatic cancer: role of LIN-28 and SET oncoprotein. PLoS One (2013) 8:e53436. 10.1371/journal.pone.0053436 23335963 PMC3546076

[102] Wu K Yang Y Zhao J Zhao S BAG3-mediated miRNA let-7g and let-7i inhibit proliferation and enhance apoptosis of human esophageal carcinoma cells by targeting the drug transporter ABCC10. Cancer Lett (2016) 371:125–33. 10.1016/j.canlet.2015.11.031 26655271

[103] Sasaki M Ikeda H Itatsu K Yamaguchi J Sawada S Minato H The overexpression of polycomb group proteins Bmi1 and EZH2 is associated with the progression and aggressive biological behavior of hepatocellular carcinoma. Lab Invest (2008) 88:873–82. 10.1038/labinvest.2008.52 18591938

[104] Xu L Beckebaum S Iacob S Wu G Kaiser GM Radtke A MicroRNA-101 inhibits human hepatocellular carcinoma progression through EZH2 downregulation and increased cytostatic drug sensitivity. J Hepatol (2014) 60:590–8. 10.1016/j.jhep.2013.10.028 24211739

[105] He H Tian W Chen H Deng Y. MicroRNA-101 sensitizes hepatocellular carcinoma cells to doxorubicin-induced apoptosis via targeting Mcl-1. Mol Med Rep (2016) 13:1923–9. 10.3892/mmr.2015.4727 26718267

[106] Nishida N Yamashita S Mimori K Sudo T Tanaka F Shibata K MicroRNA-10b is a prognostic indicator in colorectal cancer and confers resistance to the chemotherapeutic agent 5-fluorouracil in colorectal cancer cells. Ann Surg Oncol (2012) 19:3065–71. 10.1245/s10434-012-2246-1 22322955

[107] Le Gall M Chambard JC Breittmayer JP Grall D Pouyssegur J Van Obberghen-Schilling E. The p42/p44 MAP kinase pathway prevents apoptosis induced by anchorage and serum removal. Mol Biol Cell (2000) 11:1103–12. 10.1091/mbc.11.3.1103 10712523 PMC14834

[108] Mebratu Y Tesfaigzi Y. How ERK1/2 activation controls cell proliferation and cell death: is subcellular localization the answer? Cell Cycle (2009) 8:1168–75. 10.4161/cc.8.8.8147 19282669 PMC2728430

[109] Zhang Y Qu X Li C Fan Y Che X Wang X miR-103/107 modulates multidrug resistance in human gastric carcinoma by downregulating Cav-1. Tumour Biol (2015) 36:2277–85. 10.1007/s13277-014-2835-7 25407491

[110] Xiao B Guo J Miao Y Jiang Z Huan R Zhang Y Detection of miR-106a in gastric carcinoma and its clinical significance. Clin Chim Acta (2009) 400:97–102. 10.1016/j.cca.2008.10.021 18996365

[111] Wang Z Liu M Zhu H Zhang W He S Hu C miR-106a is frequently upregulated in gastric cancer and inhibits the extrinsic apoptotic pathway by targeting FAS. Mol Carcinog (2013) 52:634–46. 10.1002/mc.21899 22431000

[112] Zhang D Xiao YF Zhang JW Xie R Hu CJ Tang B miR-1182 attenuates gastric cancer proliferation and metastasis by targeting the open reading frame of hTERT. Cancer Lett (2015) 360:151–9. 10.1016/j.canlet.2015.01.044 25662441

[113] Fornari F Gramantieri L Giovannini C Veronese A Ferracin M Sabbioni S miR-122/cyclin G1 interaction modulates p53 activity and affects doxorubicin sensitivity of human hepatocarcinoma cells. Cancer Res (2009) 69:5761–7. 10.1158/0008-5472.CAN-08-4797 19584283

[114] Xu Y Xia F Ma L Shan J Shen J Yang Z MicroRNA-122 sensitizes HCC cancer cells to adriamycin and vincristine through modulating expression of MDR and inducing cell cycle arrest. Cancer Lett (2011) 310:160–9. 10.1016/j.canlet.2011.06.027 21802841

[115] Pullen TJ Xavier GD Kelsey G Rutter GA. miR-29a and miR-29b contribute to pancreatic beta-cell-specific silencing of monocarboxylate transporter 1 (Mct1). Mol Cell Biol (2011) 31:3182–94. 10.1128/MCB.01433-10 21646425 PMC3147603

[116] Hasegawa S Eguchi H Nagano H Konno M Tomimaru Y Wada H MicroRNA-1246 expression associated with CCNG2-mediated chemoresistance and stemness in pancreatic cancer. Br J Cancer (2014) 111:1572–80. 10.1038/bjc.2014.454 25117811 PMC4200094

[117] Karaayvaz M Zhai H Ju J. miR-129 promotes apoptosis and enhances chemosensitivity to 5-fluorouracil in colorectal cancer. Cell Death Dis (2013) 4:e659. 10.1038/cddis.2013.193 23744359 PMC3702282

[118] Pan YZ Zhou A Hu ZH Yu AM. Small nucleolar RNA-derived microRNA hsa-miR-1291 modulates cellular drug disposition through direct targeting of ABC transporter ABCC1. Drug Metab Dispos (2013) 41:1744–51. 10.1124/dmd.113.052092 23686318 PMC3781368

[119] Ma S Tang KH Chan YP Lee TK Kwan PS Castilho A miR-130b Promotes CD133(+) liver tumor-initiating cell growth and self-renewal via tumor protein 53-induced nuclear protein 1. Cell Stem Cell (2010) 7:694–707. 10.1016/j.stem.2010.11.010 21112564

[120] Carotenuto P Zitto D Previdi MC Raj M Fassan M Lampis A miR1307 mediates pancreatic cancer resistance to FOLFIRINOX chemotherapy by affecting response to DNA damage. Proc Am Assoc Cancer Res (2018) 59:Abstract #4977.

[121] Kano M Seki N Kikkawa N Fujimura L Hoshino I Akutsu Y miR-145, miR-133a and miR-133b: tumor-suppressive miRNAs target FSCN1 in esophageal squamous cell carcinoma. Int J Cancer (2010) 127:2804–14. 10.1002/ijc.25284 21351259

[122] Ikemura K Yamamoto M Miyazaki S Mizutani H Iwamoto T Okuda M. MicroRNA-145 post-transcriptionally regulates the expression and function of P-glycoprotein in intestinal epithelial cells. Mol Pharmacol (2013) 83:399–405. 10.1124/mol.112.081844 23166305

[123] Lee CG Mccarthy S Gruidl M Timme C Yeatman TJ. MicroRNA-147 induces a mesenchymal-to-epithelial transition (MET) and reverses EGFR inhibitor resistance. PLoS One (2014) 9:e84597. 10.1371/journal.pone.0084597 24454732 PMC3893127

[124] Valeri N Gasparini P Fabbri M Braconi C Veronese A Lovat F Modulation of mismatch repair and genomic stability by miR-155. Proc Natl Acad Sci U S A (2010) 107:6982–7. 10.1073/pnas.1002472107 20351277 PMC2872463

[125] Xia L Zhang D Du R Pan Y Zhao L Sun S miR-15b and miR-16 modulate multidrug resistance by targeting BCL2 in human gastric cancer cells. Int J Cancer (2008) 123:372–9. 10.1002/ijc.23501 18449891

[126] Fang L Li H Wang L Hu J Jin T Wang J MicroRNA-17-5p promotes chemotherapeutic drug resistance and tumour metastasis of colorectal cancer by repressing PTEN expression. Oncotarget (2014) 5:2974–87. 10.18632/oncotarget.1614 24912422 PMC4102784

[127] Yan HJ Liu WS Sun WH Wu J Ji M Wang Q miR-17-5p inhibitor enhances chemosensitivity to gemcitabine via upregulating Bim expression in pancreatic cancer cells. Dig Dis Sci (2012) 57:3160–7. 10.1007/s10620-012-2400-4 23001407

[128] Xu K Liang X Cui D Wu Y Shi W Liu J. miR-1915 inhibits Bcl-2 to modulate multidrug resistance by increasing drug-sensitivity in human colorectal carcinoma cells. Mol Carcinog (2013) 52:70–8. 10.1002/mc.21832 22121083

[129] Boni V Bitarte N Cristobal I Zarate R Rodriguez J Maiello E miR-192/miR-215 influence 5-fluorouracil resistance through Cell cycle-mediated mechanisms complementary to its post-transcriptional thymidilate synthase regulation. Mol Cancer Ther (2010) 9:2265–75. 10.1158/1535-7163.MCT-10-0061 20647341

[130] Braconi C Valeri N Gasparini P Huang N Taccioli C Nuovo G Hepatitis C virus proteins modulate microRNA expression and chemosensitivity in malignant hepatocytes. Clin Cancer Res (2010) 16:957–66. 10.1158/1078-0432.CCR-09-2123 20103677 PMC2818698

[131] Qu J Zhao L Zhang P Wang J Xu N Mi W MicroRNA-195 chemosensitizes colon cancer cells to the chemotherapeutic drug doxorubicin by targeting the first binding site of BCL2L2 mRNA. J Cell Physiol (2015) 230:535–45. 10.1002/jcp.24366 23526568

[132] Abukhdeir AM Park BH. p21 and p27: roles in carcinogenesis and drug resistance. Expert Rev Mol Med (2008) 10:e19. 10.1017/S1462399408000744 18590585 PMC2678956

[133] Fornari F Milazzo M Chieco P Negrini M Calin GA Grazi GL miR-199a-3p regulates mTOR and c-Met to influence the doxorubicin sensitivity of human hepatocarcinoma cells. Cancer Res (2010) 70:5184–93. 10.1158/0008-5472.CAN-10-0145 20501828

[134] Wang F Li T Zhang B Li H Wu Q Yang L MicroRNA-19a/b regulates multidrug resistance in human gastric cancer cells by targeting PTEN. Biochem Biophys Res Commun (2013) 434:688–94. 10.1016/j.bbrc.2013.04.010 23603256

[135] Yu J Ohuchida K Mizumoto K Sato N Kayashima T Fujita H MicroRNA, hsa-miR-200c, is an independent prognostic factor in pancreatic cancer and its upregulation inhibits pancreatic cancer invasion but increases cell proliferation. Mol Cancer (2010) 9:169. 10.1186/1476-4598-9-169 20579395 PMC2909980

[136] Zhou Y Wan G Spizzo R Ivan C Mathur R Hu X miR-203 induces oxaliplatin resistance in colorectal cancer cells by negatively regulating ATM kinase. Mol Oncol (2014) 8:83–92. 10.1016/j.molonc.2013.09.004 24145123 PMC4124530

[137] Singh S Chitkara D Kumar V Behrman SW Mahato RI. miRNA profiling in pancreatic cancer and restoration of chemosensitivity. Cancer Lett (2013) 334:211–20. 10.1016/j.canlet.2012.10.008 23073476

[138] Valeri N Gasparini P Braconi C Paone A Lovat F Fabbri M MicroRNA-21 induces resistance to 5-fluorouracil by down-regulating human DNA MutS homolog 2 (hMSH2). Proc Natl Acad Sci U S A (2010) 107:21098–103. 10.1073/pnas.1015541107 21078976 PMC3000294

[139] Yu Y Sarkar FH Majumdar AP. Down-regulation of miR-21 induces differentiation of chemoresistant colon cancer cells and enhances susceptibility to therapeutic regimens. Transl Oncol (2013) 6:180–6. 10.1593/tlo.12397 23544170 PMC3610548

[140] Giovannetti E Funel N Peters GJ Del Chiaro M Erozenci LA Vasile E MicroRNA-21 in pancreatic cancer: correlation with clinical outcome and pharmacologic aspects underlying its role in the modulation of gemcitabine activity. Cancer Res (2010) 70:4528–38. 10.1158/0008-5472.CAN-09-4467 20460539

[141] Dong J Zhao YP Zhou L Zhang TP Chen G Bcl-2 upregulation induced by miR-21 via a direct interaction is associated with apoptosis and chemoresistance in MIA PaCa-2 pancreatic cancer cells. Arch Med Res (2011) 42:8–14. 10.1016/j.arcmed.2011.01.006 21376256

[142] Meng F Henson R Wehbe-Janek H Ghoshal K Jacob ST Patel T. MicroRNA-21 regulates expression of the PTEN tumor suppressor gene in human hepatocellular cancer. Gastroenterology (2007) 133:647–58. 10.1053/j.gastro.2007.05.022 17681183 PMC4285346

[143] Zhang BG Li JF Yu BQ Zhu ZG Liu BY Yan M. MicroRNA-21 promotes tumor proliferation and invasion in gastric cancer by targeting PTEN. Oncol Rep (2012) 27:1019–26. 10.3892/or.2012.1645 22267008 PMC3583594

[144] Yang SM Huang C Li XF Yu MZ He Y Li J miR-21 confers cisplatin resistance in gastric cancer cells by regulating PTEN. Toxicology (2013) 306:162–8. 10.1016/j.tox.2013.02.014 23466500

[145] Frampton AE Castellano L Colombo T Giovannetti E Krell J Jacob J MicroRNAs cooperatively inhibit a network of tumor suppressor genes to promote pancreatic tumor growth and progression. Gastroenterology (2014) 146:268–77.e18. 10.1053/j.gastro.2013.10.010 24120476

[146] Frampton AE Giovannetti E Jamieson NB Krell J Gall TM Stebbing J A microRNA meta-signature for pancreatic ductal adenocarcinoma. Expert Rev Mol Diagn (2014) 14:267–71. 10.1586/14737159.2014.893192 24575833

[147] Maftouh M Avan A Funel N Frampton AE Fiuji H Pelliccioni S miR-211 modulates gemcitabine activity through downregulation of ribonucleotide reductase and inhibits the invasive behavior of pancreatic cancer cells. Nucleosides Nucleotides Nucleic Acids (2014) 33:384–93. 10.1080/15257770.2014.891741 24940696

[148] Wang L Wang YM Xu S Wang WG Chen Y Mao JY MicroRNA-215 is upregulated by treatment with adriamycin and leads to the chemoresistance of hepatocellular carcinoma cells and tissues. Mol Med Rep (2015) 12:5274–80. 10.3892/mmr.2015.4012 26135967

[149] Deng Y Huang Z Xu Y Jin J Zhuo W Zhang C miR-215 modulates gastric cancer cell proliferation by targeting RB1. Cancer Lett (2014) 342:27–35. 10.1016/j.canlet.2013.08.033 23981575

[150] Xu YJ Fan Y. miR-215/192 participates in gastric cancer progression. Clin Transl Oncol (2015) 17:34–40. 10.1007/s12094-014-1194-6 24981590

[151] Li J Zhang Y Zhao J Kong F Chen Y. Overexpression of miR-22 reverses paclitaxel-induced chemoresistance through activation of PTEN signaling in p53-mutated colon cancer cells. Mol Cell Biochem (2011) 357:31–8. 10.1007/s11010-011-0872-8 21594648

[152] Wang Y Zhao Y Herbst A Kalinski T Qin J Wang X miR-221 mediates chemoresistance of esophageal adenocarcinoma by direct targeting of DKK2 expression. Ann Surg (2016) 264:804–14. 10.1097/SLA.0000000000001928 27501171

[153] Ma J Fang B Zeng F Ma C Pang H Cheng L Down-regulation of miR-223 reverses epithelial-mesenchymal transition in gemcitabine-resistant pancreatic cancer cells. Oncotarget (2015) 6:1740–9. 10.18632/oncotarget.2714 25638153 PMC4359328

[154] Zhou X Jin W Jia H Yan J Zhang G. miR-223 promotes the cisplatin resistance of human gastric cancer cells via regulating cell cycle by targeting FBXW7. J Exp Clin Cancer Res (2015) 34:28. 10.1186/s13046-015-0145-6 25888377 PMC4387683

[155] Amankwatia EB Chakravarty P Carey FA Weidlich S Steele RJ Munro AJ MicroRNA-224 is associated with colorectal cancer progression and response to 5-fluorouracil-based chemotherapy by KRAS-dependent and -independent mechanisms. Br J Cancer (2015) 112:1480–90. 10.1038/bjc.2015.125 25919696 PMC4453675

[156] Li X Li X Liao D Wang X Wu Z Nie J Elevated microRNA-23a expression enhances the chemoresistance of colorectal cancer cells with microsatellite instability to 5-fluorouracil by directly targeting ABCF1. Curr Protein Pept Sci (2015) 16:301–9. 10.2174/138920371604150429153309 25929864

[157] Zhao H Wang Y Yang L Jiang R Li W miR-25 promotes gastric cancer cells growth and motility by targeting RECK. Mol Cell Biochem (2014) 385:207–13. 10.1007/s11010-013-1829-x 24078004

[158] Gong J Cui Z Li L Ma Q Wang Q Gao Y MicroRNA-25 promotes gastric cancer proliferation, invasion, and migration by directly targeting F-box and WD-40 domain protein 7, FBXW7. Tumour Biol (2015) 36:7831–40. 10.1007/s13277-015-3510-3 25944166

[159] Li BS Zuo QF Zhao YL Xiao B Zhuang Y Mao XH MicroRNA-25 promotes gastric cancer migration, invasion and proliferation by directly targeting transducer of ERBB2, 1 and correlates with poor survival. Oncogene (2015) 34:2556–65. 10.1038/onc.2014.214 25043310

[160] He J Qi H Chen F Cao C. MicroRNA-25 contributes to cisplatin resistance in gastric cancer cells by inhibiting forkhead box O3a. Oncol Lett (2017) 14:6097–102. 10.3892/ol.2017.6982 29113252 PMC5661442

[161] Fan Y Dutta J Gupta N Fan G Gelinas C. Regulation of programmed cell death by NF-kappaB and its role in tumorigenesis and therapy. Adv Exp Med Biol (2008) 615:223–50. 10.1007/978-1-4020-6554-5_11 18437897

[162] Zhao N Wang R Zhou L Zhu Y Gong J Zhuang SM MicroRNA-26b suppresses the NF-kappaB signaling and enhances the chemosensitivity of hepatocellular carcinoma cells by targeting TAK1 and TAB 3. Mol Cancer (2014) 13:35 10.1186/1476-4598-13-35 24565101 PMC3938074

[163] Offer SM Butterfield GL Jerde CR Fossum CC Wegner NJ Diasio RB. MicroRNAs miR-27a and miR-27b directly regulate liver dihydropyrimidine dehydrogenase expression through two conserved binding sites. Mol Cancer Ther (2014) 13:742–51. 10.1158/1535-7163.MCT-13-0878 24401318 PMC3954441

[164] An X Sarmiento C Tan T Zhu H. Regulation of multidrug resistance by microRNAs in anti-cancer therapy. Acta Pharm Sin B (2017) 7:38–51. 10.1016/j.apsb.2016.09.002 28119807 PMC5237711

[165] Pan YZ Gao WQ Yu AM. MicroRNAs regulate CYP3A4 expression via direct and indirect targeting. Drug Metab Dispos (2009) 37:2112–7. 10.1124/dmd.109.027680 19581388 PMC2769037

[166] Xu K Liang X Shen K Cui DL Zheng YH Xu JH miR-297 modulates multidrug resistance in human colorectal carcinoma by down-regulating MRP-2. Biochem J (2012) 446:291–300. 10.1042/BJ20120386 22676135

[167] Wang CJ Stratmann J Zhou ZG Sun XF Suppression of microRNA-31 increases sensitivity to 5-FU at an early stage, and affects cell migration and invasion in HCT-116 colon cancer cells. BMC Cancer (2010) 10:616 10.1186/1471-2407-10-616 21062447 PMC2994822

[168] Cekaite L Rantala JK Bruun J Guriby M Agesen TH Danielsen SA miR-9, -31, and -182 deregulation promote proliferation and tumor cell survival in colon cancer. Neoplasia (2012) 14:868–79. 10.1593/neo.121094 23019418 PMC3459282

[169] Wan LY Deng J Xiang XJ Zhang L Yu F Chen J miR-320 enhances the sensitivity of human colon cancer cells to chemoradiotherapy in vitro by targeting FOXM1. Biochem Biophys Res Commun (2015) 457:125–32. 10.1016/j.bbrc.2014.11.039 25446103

[170] Vishnubalaji R Hamam R Yue S Al-Obeed O Kassem M Liu FF MicroRNA-320 suppresses colorectal cancer by targeting SOX4, FOXM1, and FOXQ1. Oncotarget (2016) 7:35789–802. 10.18632/oncotarget.8937 27119506 PMC5094962

[171] Han J Li J Tang K Zhang H Guo B Hou N miR-338-3p confers 5-fluorouracil resistance in p53 mutant colon cancer cells by targeting the mammalian target of rapamycin. Exp Cell Res (2017) 360:328–36. 10.1016/j.yexcr.2017.09.023 28928082

[172] Wang BD Kline CL Pastor DM Olson TL Frank B Luu T Prostate apoptosis response protein 4 sensitizes human colon cancer cells to chemotherapeutic 5-FU through mediation of an NF kappaB and microRNA network. Mol Cancer (2010) 9:98 10.1186/1476-4598-9-98 20433755 PMC2883962

[173] Nie J Liu L Zheng W Chen L Wu X Xu Y microRNA-365, down-regulated in colon cancer, inhibits cell cycle progression and promotes apoptosis of colon cancer cells by probably targeting Cyclin D1 and Bcl-2. Carcinogenesis (2012) 33:220–5. 10.1093/carcin/bgr245 22072615

[174] Schreiber R Mezencev R Matyunina LV Mcdonald JF. Evidence for the role of microRNA 374b in acquired cisplatin resistance in pancreatic cancer cells. Cancer Gene Ther (2016) 23:241–5. 10.1038/cgt.2016.23 27229158 PMC5007605

[175] Mohri T Nakajima M Fukami T Takamiya M Aoki Y Yokoi T. Human CYP2E1 is regulated by miR-378. Biochem Pharmacol (2010) 79:1045–52. 10.1016/j.bcp.2009.11.015 19945440

[176] Tan S Shi H Ba M Lin S Tang H Zeng X miR-409-3p sensitizes colon cancer cells to oxaliplatin by inhibiting beclin-1-mediated autophagy. Int J Mol Med (2016) 37:1030–8. 10.3892/ijmm.2016.2492 26935807

[177] Bitarte N Bandres E Boni V Zarate R Rodriguez J Gonzalez-Huarriz M MicroRNA-451 is involved in the self-renewal, tumorigenicity, and chemoresistance of colorectal cancer stem cells. Stem Cells (2011) 29:1661–71. 10.1002/stem.741 21948564

[178] Chai J Dong W Xie C Wang L Han DL Wang S MicroRNA-494 sensitizes colon cancer cells to fluorouracil through regulation of DPYD. IUBMB Life (2015) 67:191–201. 10.1002/iub.1361 25873402

[179] Xu K Chen G Qiu Y Yuan Z Li H Yuan X miR-503-5p confers drug resistance by targeting PUMA in colorectal carcinoma. Oncotarget (2017) 8:21719–32. 10.18632/oncotarget.15559 28423513 PMC5400618

[180] Shang Y Zhang Z Liu Z Feng B Ren G Li K miR-508-5p regulates multidrug resistance of gastric cancer by targeting ABCB1 and ZNRD1. Oncogene (2014) 33:3267–76. 10.1038/onc.2013.297 23893241

[181] Fornari F Milazzo M Chieco P Negrini M Marasco E Capranico G In hepatocellular carcinoma miR-519d is up-regulated by p53 and DNA hypomethylation and targets CDKN1A/p21, PTEN, AKT3 and TIMP2. J Pathol (2012) 227:275–85. 10.1002/path.3995 22262409

[182] Yang G Jiang O Ling DQ Jiang XY Yuan PZ Zeng G MicroRNA-522 reverses drug resistance of doxorubicin-induced HT29 colon cancer cell by targeting ABCB5. Mol Med Rep (2015) 12:3930–6. 10.3892/mmr.2015.3890 26043974

[183] Dalmasso G Hang TTN Yan YT Laroui H Charania MA Obertone TS MicroRNA-92b regulates expression of the oligopeptide transporter PepT1 in intestinal epithelial cells. Am J Physiol Gastrointest Liver Physiol (2011) 300:G52–9. 10.1152/ajpgi.00394.2010 21030610 PMC3025505

[184] Zhang JX Xu Y Gao Y Chen C Zheng ZS Yun M Decreased expression of miR-939 contributes to chemoresistance and metastasis of gastric cancer via dysregulation of SLC34A2 and Raf/MEK/ERK pathway. Mol Cancer (2017) 16:18. 10.1186/s12943-017-0586-y 28114937 PMC5259972

[185] Kim SA Kim I Yoon SK Lee EK Kuh HJ. Indirect modulation of sensitivity to 5-fluorouracil by microRNA-96 in human colorectal cancer cells. Arch Pharm Res (2015) 38:239–48. 10.1007/s12272-014-0528-9 25502560

[186] Persson H Kvist A Vallon-Christersson J Medstrand P Borg A Rovira C. The non-coding RNA of the multidrug resistance-linked vault particle encodes multiple regulatory small RNAs. Nat Cell Biol (2009) 11:1268–71. 10.1038/ncb1972 19749744

[187] Mossink MH Van Zon A Scheper RJ Sonneveld P Wiemer EAC. Vaults: a ribonucleoprotein particle involved in drug resistance? Oncogene (2003) 22:7458–67. 10.1038/sj.onc.1206947 14576851

[188] Gopinath SC Wadhwa R Kumar PK. Expression of noncoding vault RNA in human malignant cells and its importance in mitoxantrone resistance. Mol Cancer Res (2010) 8:1536–46. 10.1158/1541-7786.MCR-10-0242 20881010

[189] Geisler S Coller J. RNA in unexpected places: long non-coding RNA functions in diverse cellular contexts. Nat Rev Mol Cell Biol (2013) 14:699–712. 10.1038/nrm3679 24105322 PMC4852478

[190] Taal BG Audisio RA Bleiberg H Blijham GH Neijt JP Veenhof CH Phase II trial of mitomycin C (MMC) in advanced gallbladder and biliary tree carcinoma. An EORTC Gastrointestinal Tract Cancer Cooperative Group Study. Ann Oncol (1993) 4:607–9. 10.1093/oxfordjournals.annonc.a058597 8363992

[191] Eddy SR. Non-coding RNA genes and the modern RNA world. Nat Rev Genet (2001) 2:919–29. 10.1038/35103511 11733745

[192] He L Hannon GJ MicroRNAs: small RNAs with a big role in gene regulation. Nat Rev Genet (2004) 5:522–31. 10.1038/nrg1379 15211354

[193] Guttman M Amit I Garber M French C Lin MF Feldser D Chromatin signature reveals over a thousand highly conserved large non-coding RNAs in mammals. Nature (2009) 458:223–7. 10.1038/nature07672 19182780 PMC2754849

[194] Langenberger D Bermudez-Santana C Hertel J Hoffmann S Khaitovich P Stadler PF. Evidence for human microRNA-offset RNAs in small RNA sequencing data. Bioinformatics (2009) 25:2298–301. 10.1093/bioinformatics/btp419 19584066

[195] Taft RJ Glazov EA Cloonan N Simons C Stephen S Faulkner GJ Tiny RNAs associated with transcription start sites in animals. Nat Genet (2009) 41:572–8. 10.1038/ng0709-859a 19377478

[196] Taft RJ Glazov EA Lassmann T Hayashizaki Y Carninci P Mattick JS Small RNAs derived from snoRNAs. RNA (2009) 15:1233–40. 10.1261/rna.1528909 19474147 PMC2704076

[197] Wilusz JE Sunwoo H Spector DL. Long noncoding RNAs: functional surprises from the RNA world. Genes Dev (2009) 23:1494–504. 10.1101/gad.1800909 19571179 PMC3152381

[198] Choudhuri S. Small noncoding RNAs: biogenesis, function, and emerging significance in toxicology. J Biochem Mol Toxicol (2010) 24:195–216. 10.1002/jbt.20325 20143452

[199] Ling H Fabbri M Calin GA. MicroRNAs and other non-coding RNAs as targets for anticancer drug development. Nat Rev Drug Discov (2013) 12:847–65. 10.1038/nrd4140 24172333 PMC4548803

[200] Claycomb JM. Ancient endo-siRNA pathways reveal new tricks. Curr Biol (2014) 24:R703–15. 10.1016/j.cub.2014.06.009 25093565

[201] Guo JU Agarwal V Guo H Bartel DP. Expanded identification and characterization of mammalian circular RNAs. Genome Biol (2014) 15:409. 10.1186/s13059-014-0409-z 25070500 PMC4165365

[202] An Y Furber KL Ji S Pseudogenes regulate parental gene expression via ceRNA network. J Cell Mol Med (2016) 21(1):185–92. 10.1111/jcmm.12952 27561207 PMC5192809

[203] Azlan A Dzaki N Azzam G. Argonaute: the executor of small RNA function. J Genet Genomics (2016) 43:481–94. 10.1016/j.jgg.2016.06.002 27569398

[204] Beermann J Piccoli MT Viereck J Thum T. Non-coding RNAs in development and disease: background, mechanisms, and therapeutic approaches. Physiol Rev (2016) 96:1297–325. 10.1152/physrev.00041.2015 27535639

[205] De Almeida RA Fraczek MG Parker S Delneri D O’keefe RT. Non-coding RNAs and disease: the classical ncRNAs make a comeback. Biochem Soc Trans (2016) 44:1073–8. 10.1042/BST20160089 27528754 PMC6042638

[206] Evans JR Feng FY Chinnaiyan AM. The bright side of dark matter: lncRNAs in cancer. J Clin Invest (2016) 126:2775–82. 10.1172/JCI84421 27479746 PMC4966302

[207] Geiger J Dalgaard LT Interplay of mitochondrial metabolism and microRNAs. Cell Mol Life Sci (2016) 74(4):631–46. 10.1007/s00018-016-2342-7 27563705 PMC11107739

[208] Granados-Riveron JT Aquino-Jarquin G. The complexity of the translation ability of circRNAs. Biochim Biophys Acta (2016) 1859:1245–51. 10.1016/j.bbagrm.2016.07.009 27449861

[209] Khurana E Fu Y Chakravarty D Demichelis F Rubin MA Gerstein M Role of non-coding sequence variants in cancer. Nat Rev Genet (2016) 17:93–108. 10.1038/nrg.2015.17 26781813

[210] Qi P Zhou XY Du X. Circulating long non-coding RNAs in cancer: current status and future perspectives. Mol Cancer (2016) 15:39. 10.1186/s12943-016-0524-4 27189224 PMC4869386

[211] Quinn JJ Chang HY. Unique features of long non-coding RNA biogenesis and function. Nat Rev Genet (2016) 17:47–62. 10.1038/nrg.2015.10 26666209

[212] Guttman M Donaghey J Carey BW Garber M Grenier JK Munson G lincRNAs act in the circuitry controlling pluripotency and differentiation. Nature (2011) 477:295–300. 10.1038/nature10398 21874018 PMC3175327

[213] Sauvageau M Goff LA Lodato S Bonev B Groff AF Gerhardinger C Multiple knockout mouse models reveal lincRNAs are required for life and brain development. Elife (2013) 2:e01749. 10.7554/eLife.01749 24381249 PMC3874104

[214] Herriges MJ Swarr DT Morley MP Rathi KS Peng T Stewart KM Long noncoding RNAs are spatially correlated with transcription factors and regulate lung development. Genes Dev (2014) 28:1363–79. 10.1101/gad.238782.114 24939938 PMC4066405

[215] Li G Zhang H Wan X Yang X Zhu C Wang A Long noncoding RNA plays a key role in metastasis and prognosis of hepatocellular carcinoma. Biomed Res Int (2014) 2014:780521 10.1155/2014/780521 24757675 PMC3976793

[216] Ounzain S Pezzuto I Micheletti R Burdet F Sheta R Nemir M Functional importance of cardiac enhancer-associated noncoding RNAs in heart development and disease. J Mol Cell Cardiol (2014) 76:55–70. 10.1016/j.yjmcc.2014.08.009 25149110 PMC4445080

[217] Moran VA Perera RJ Khalil AM. Emerging functional and mechanistic paradigms of mammalian long non-coding RNAs. Nucleic Acids Res (2012) 40:6391–400. 10.1093/nar/gks296 22492512 PMC3413108

[218] Kornienko AE Guenzl PM Barlow DP Pauler FM. Gene regulation by the act of long non-coding RNA transcription. BMC Biol (2013) 11:59. 10.1186/1741-7007-11-59 23721193 PMC3668284

[219] Han P Chang CP. Long non-coding RNA and chromatin remodeling. RNA Biol (2015) 12:1094–8. 10.1080/15476286.2015.1063770 26177256 PMC4829272

[220] Yoon JH Abdelmohsen K Gorospe M. Functional interactions among microRNAs and long noncoding RNAs. Semin Cell Dev Biol (2014) 34:9–14. 10.1016/j.semcdb.2014.05.015 24965208 PMC4163095

[221] Fabbri M Valeri N Calin GA. MicroRNAs and genomic variations: from proteus tricks to prometheus gift. Carcinogenesis (2009) 30:912–7. 10.1093/carcin/bgp063 19293341

[222] Valeri N Vannini I Fanini F Calore F Adair B Fabbri M. Epigenetics, miRNAs, and human cancer: a new chapter in human gene regulation. Mamm Genome (2009) 20:573–80. 10.1007/s00335-009-9206-5 19697081

[223] Winter J Jung S Keller S Gregory RI Diederichs S. Many roads to maturity: microRNA biogenesis pathways and their regulation. Nat Cell Biol (2009) 11:228–34. 10.1038/ncb0309-228 19255566

[224] Macfarlane LA Murphy PR MicroRNA: biogenesis, function and role in cancer. Curr Genomics (2010) 11:537–61. 10.2174/138920210793175895 21532838 PMC3048316

[225] Pasquinelli AE. MicroRNAs and their targets: recognition, regulation and an emerging reciprocal relationship. Nat Rev Genet (2012) 13:271–82. 10.1038/nrg3162 22411466

[226] Von Schack D Agostino MJ Murray BS Li Y Reddy PS Chen J Dynamic changes in the microRNA expression profile reveal multiple regulatory mechanisms in the spinal nerve ligation model of neuropathic pain. PLoS One (2011) 6:e17670. 10.1371/journal.pone.0017670 21423802 PMC3056716

[227] Lagos-Quintana M Rauhut R Yalcin A Meyer J Lendeckel W Tuschl T. Identification of tissue-specific microRNAs from mouse. Curr Biol (2002) 12:735–9. 10.1016/S0960-9822(02)00809-6 12007417

[228] Lim LP Lau NC Garrett-Engele P Grimson A Schelter JM Castle J Microarray analysis shows that some microRNAs downregulate large numbers of target mRNAs. Nature (2005) 433:769–73. 10.1038/nature03315 15685193

[229] Murakami Y Yasuda T Saigo K Urashima T Toyoda H Okanoue T Comprehensive analysis of microRNA expression patterns in hepatocellular carcinoma and non-tumorous tissues. Oncogene (2006) 25:2537–45. 10.1038/sj.onc.1209283 16331254

[230] Mitchell PS Parkin RK Kroh EM Fritz BR Wyman SK Pogosova-Agadjanyan EL Circulating microRNAs as stable blood-based markers for cancer detection. Proc Natl Acad Sci U S A (2008) 105:10513–8. 10.1073/pnas.0804549105 18663219 PMC2492472

[231] O’connell RM Rao DS Chaudhuri AA Baltimore D. Physiological and pathological roles for microRNAs in the immune system. Nat Rev Immunol (2010) 10:111–22. 10.1038/nri2708 20098459

[232] Esteller M. Non-coding RNAs in human disease. Nat Rev Genet (2011) 12:861–74. 10.1038/nrg3074 22094949

[233] Ha TY MicroRNAs in human diseases: from cancer to cardiovascular disease. Immune Netw (2011) 11:135–54. 10.4110/in.2011.11.3.135 21860607 PMC3153666

[234] Ha TY MicroRNAs in human diseases: from lung, liver and kidney diseases to infectious disease, sickle cell disease and endometrium disease. Immune Netw (2011) 11:309–23. 10.4110/in.2011.11.6.309 22346770 PMC3275699

[235] Ha TY The role of microRNAs in regulatory T cells and in the immune response. Immune Netw (2011) 11:11–41. 10.4110/in.2011.11.1.11 21494372 PMC3072673

[236] Grasedieck S Scholer N Bommer M Niess JH Tumani H Rouhi A Impact of serum storage conditions on microRNA stability. Leukemia (2012) 26:2414–6. 10.1038/leu.2012.106 22504138

[237] Iorio MV Croce CM. MicroRNA involvement in human cancer. Carcinogenesis (2012) 33:1126–33. 10.1093/carcin/bgs140 22491715 PMC3514864

[238] Acunzo M Romano G Wernicke D Croce CM MicroRNA and cancer – a brief overview. Adv Biol Regul (2015) 57:1–9. 10.1016/j.jbior.2014.09.013 25294678

[239] Balatti V Pekarky Y Croce CM. Role of microRNA in chronic lymphocytic leukemia onset and progression. J Hematol Oncol (2015) 8:12. 10.1186/s13045-015-0112-x 25886051 PMC4336680

[240] Gardiner AS Twiss JL Perrone-Bizzozero NI. Competing interactions of RNA-binding proteins, microRNAs, and their targets control neuronal development and function. Biomolecules (2015) 5:2903–18. 10.3390/biom5042903 26512708 PMC4693262

[241] Gottesman MM Fojo T Bates SE. Multidrug resistance in cancer: role of ATP-dependent transporters. Nat Rev Cancer (2002) 2:48–58. 10.1038/nrc706 11902585

[242] Longley DB Johnston PG. Molecular mechanisms of drug resistance. J Pathol (2005) 205:275–92. 10.1002/path.1706 15641020

[243] Rodrigues AS Dinis J Gromicho M Martins C Laires A Rueff J Genomics and cancer drug resistance. Curr Pharm Biotechnol (2012) 13:651–73. 10.2174/138920112799857549 22122479

[244] Holohan C Van Schaeybroeck S Longley DB Johnston PG Cancer drug resistance: an evolving paradigm. Nat Rev Cancer (2013) 13:714–26. 10.1038/nrc3599 24060863

[245] Michael M Doherty MM. Tumoral drug metabolism: overview and its implications for cancer therapy. J Clin Oncol (2005) 23:205–29. 10.1200/JCO.2005.02.120 15625375

[246] Park GR Sedation and analgesia-which way is best? Br J Anaesth (2001) 87:183–5. 10.1093/bja/87.2.183 11493485

[247] Shimada T Iwasaki M Martin MV Guengerich FP. Human liver microsomal cytochrome P-450 enzymes involved in the bioactivation of procarcinogens detected by umu gene response in *Salmonella typhimurium* TA 1535/pSK1002. Cancer Res (1989) 49:3218–28. 2655891

[248] Guengerich FP Shimada T Oxidation of toxic and carcinogenic chemicals by human cytochrome P-450 enzymes. Chem Res Toxicol (1991) 4:391–407. 10.1021/tx00022a001 1912325

[249] Shea TC Kelley SL Henner WD Identification of an anionic form of glutathione transferase present in many human-tumors and human-tumor cell-lines. Cancer Res (1988) 48:527–33. 3275499

[250] Mclellan LI Wolf CR Glutathione and glutathione-dependent enzymes in cancer drug resistance. Drug Resist Updat (1999) 2:153–64. 10.1054/drup.1999.0083 11504486

[251] Dean M Hamon Y Chimini G. The human ATP-binding cassette (ABC) transporter superfamily. J Lipid Res (2001) 42:1007–17. 10.1101/gr.184901 11441126

[252] Kathawala RJ Gupta P Ashby CR Jr Chen ZS. The modulation of ABC transporter-mediated multidrug resistance in cancer: a review of the past decade. Drug Resist Updat (2015) 18:1–17. 10.1016/j.drup.2014.11.002 25554624

[253] Lin L Yee SW Kim RB Giacomini KM. SLC transporters as therapeutic targets: emerging opportunities. Nat Rev Drug Discov (2015) 14:543–60. 10.1038/nrd4626 26111766 PMC4698371

[254] Colas C Ung PMU Schlessinger A. SLC transporters: structure, function, and drug discovery. Medchemcomm (2016) 7:1069–81. 10.1039/C6MD00005C 27672436 PMC5034948

[255] Haenisch S Werk AN Cascorbi I. MicroRNAs and their relevance to ABC transporters. Br J Clin Pharmacol (2014) 77:587–96. 10.1111/bcp.12251 24645868 PMC3971975

[256] Ikemura K Iwamoto T Okuda M. MicroRNAs as regulators of drug transporters, drug-metabolizing enzymes, and tight junctions: implication for intestinal barrier function. Pharmacol Ther (2014) 143:217–24. 10.1016/j.pharmthera.2014.03.002 24631272

[257] Munoz M Henderson M Haber M Norris M. Role of the MRP1/ABCC1 multidrug transporter protein in cancer. IUBMB Life (2007) 59:752–7. 10.1080/15216540701736285 18085475

[258] Tu MJ Pan YZ Qiu JX Kim EJH Yu AM Impact and mechanistic role of MicroRNA-1291 on pancreatic tumorigenesis. J Clin Oncol (2016) 34:243 10.1200/jco.2016.34.4_suppl.243

[259] Yang T Zheng ZM Li XN Li ZF Wang Y Geng YF miR-223 modulates multidrug resistance via downregulation of ABCB1 in hepatocellular carcinoma cells. Exp Biol Med (2013) 238:1024–32. 10.1177/1535370213497321 23925649

[260] Peterson LW Artis D. Intestinal epithelial cells: regulators of barrier function and immune homeostasis. Nat Rev Immunol (2014) 14:141–53. 10.1038/nri3608 24566914

[261] Mckenna LB Schug J Vourekas A Mckenna JB Bramswig NC Friedman JR MicroRNAs control intestinal epithelial differentiation, architecture, and barrier function. Gastroenterology (2010) 139(5):1654–64, 1664.e1. 10.1053/j.gastro.2010.07.040 20659473 PMC3156097

[262] Tsuchiya Y Nakajima M Takagi S Taniya T Yokoi T MicroRNA regulates the expression of human cytochrome P4501B1. Cancer Res (2006) 66:9090–8. 10.1158/0008-5472.CAN-06-1403 16982751

[263] Koturbash I Beland FA Pogribny IP. Role of microRNAs in the regulation of drug metabolizing and transporting genes and the response to environmental toxicants. Expert Opin Drug Metab Toxicol (2012) 8:597–606. 10.1517/17425255.2012.673587 22435483

[264] Rendic S Guengerich FP. Survey of human oxidoreductases and cytochrome P450 enzymes involved in the metabolism of xenobiotic and natural chemicals. Chem Res Toxicol (2015) 28:38–42. 10.1021/tx500444e 25485457 PMC4303333

[265] Zhou J Wen Q Li SF Zhang YF Gao N Tian X Significant change of cytochrome P450s activities in patients with hepatocellular carcinoma. Oncotarget (2016) 7:50612–23. 10.18632/oncotarget.9437 27203676 PMC5226607

[266] Helleday T Petermann E Lundin C Hodgson B Sharma RA. DNA repair pathways as targets for cancer therapy. Nat Rev Cancer (2008) 8:193–204. 10.1038/nrc2342 18256616

[267] Hanahan D Weinberg RA Hallmarks of cancer: the next generation. Cell (2011) 144:646–74. 10.1016/j.cell.2011.02.013 21376230

[268] Hoeijmakers JHJ. Genome maintenance mechanisms for preventing cancer. Nature (2001) 411:366–74. 10.1038/35077232 11357144

[269] Harper JW Elledge SJ. The DNA damage response: ten years after. Mol Cell (2007) 28:739–45. 10.1016/j.molcel.2007.11.015 18082599

[270] Jackson SP Bartek J. The DNA-damage response in human biology and disease. Nature (2009) 461:1071–8. 10.1038/nature08467 19847258 PMC2906700

[271] Pearl LH Schierz AC Ward SE Al-Lazikani B Pearl FMG. Therapeutic opportunities within the DNA damage response. Nat Rev Cancer (2015) 15:166–80. 10.1038/nrc3891 25709118

[272] Pothof J Verkaik NS Van Ijcken W Wiemer EAC Ta VTB Van Der Horst GTJ MicroRNA-mediated gene silencing modulates the UV-induced DNA-damage response. EMBO J (2009) 28:2090–9. 10.1038/emboj.2009.156 19536137 PMC2718280

[273] Wouters MD Van Gent DC Hoeijmakers JHJ Pothof J. MicroRNAs, the DNA damage response and cancer. Mutat Res (2011) 717:54–66. 10.1016/j.mrfmmm.2011.03.012 21477600

[274] Chowdhury D Choi YE Brault ME DNA DAMAGE – OPINION charity begins at home: non-coding RNA functions in DNA repair. Nat Rev Mol Cell Biol (2013) 14:181–9. 10.1038/nrm3523 23385724 PMC3904369

[275] Sharma V Misteli T. Non-coding RNAs in DNA damage and repair. FEBS Lett (2013) 587:1832–9. 10.1016/j.febslet.2013.05.006 23684639 PMC3710463

[276] Bottai G Pasculli B Calin GA Santarpia L. Targeting the microRNA-regulating DNA damage/repair pathways in cancer. Expert Opin Biol Ther (2014) 14:1667–83. 10.1517/14712598.2014.950650 25190496

[277] Niinuma T Suzuki H Nojima M Nosho K Yamamoto H Takamaru H Upregulation of miR-196a and HOTAIR drive malignant character in gastrointestinal stromal tumors. Cancer Res (2012) 72:1126–36. 10.1158/0008-5472.CAN-11-1803 22258453

[278] Waldman T Kinzler KW Vogelstein B P21 is necessary for the P53-mediated G(1) arrest in human cancer-cells. Cancer Res (1995) 55:5187–90. 7585571

[279] Bunz F Dutriaux A Lengauer C Waldman T Zhou S Brown JP Requirement for p53 and p21 to sustain G(2) arrest after DNA damage. Science (1998) 282:1497–501. 10.1126/science.282.5393.1497 9822382

[280] Choy KR Watters DJ. Neurodegeneration in ataxia-telangiectasia: multiple roles of ATM kinase in cellular homeostasis. Dev Dyn (2018) 247:33–46. 10.1002/dvdy.24522 28543935

[281] Sacconi A Biagioni F Canu V Mori F Di Benedetto A Lorenzon L miR-204 targets Bcl-2 expression and enhances responsiveness of gastric cancer. Cell Death Dis (2012) 3:e423. 10.1038/cddis.2012.160 23152059 PMC3542596

[282] Huang H Tindall DJ. FOXO factors: a matter of life and death. Future Oncol (2006) 2:83–9. 10.2217/14796694.2.1.83 16556075

[283] Nho RS Hergert P. FoxO3a and disease progression. World J Biol Chem (2014) 5:346–54. 10.4331/wjbc.v5.i3.346 25225602 PMC4160528

[284] Moriyama T Ohuchida K Mizumoto K Yu J Sato N Nabae T MicroRNA-21 modulates biological functions of pancreatic cancer cells including their proliferation, invasion, and chemoresistance. Mol Cancer Ther (2009) 8:1067–74. 10.1158/1535-7163.MCT-08-0592 19435867

[285] Park JK Lee EJ Esau C Schmittgen TD. Antisense inhibition of microRNA-21 or -221 arrests cell cycle, induces apoptosis, and sensitizes the effects of gemcitabine in pancreatic adenocarcinoma. Pancreas (2009) 38:e190–9. 10.1097/MPA.0b013e3181ba82e1 19730150

[286] Donahue TR Nguyen AH Moughan J Li L Tatishchev S Toste P Stromal microRNA-21 levels predict response to 5-fluorouracil in patients with pancreatic cancer. J Surg Oncol (2014) 110:952–9. 10.1002/jso.23750 25132574 PMC4232442

[287] Zhang XJ Ye H Zeng CW He B Zhang H Chen YQ. Dysregulation of miR-15a and miR-214 in human pancreatic cancer. J Hematol Oncol (2010) 3:46. 10.1186/1756-8722-3-46 21106054 PMC3002909

[288] Khan KH Yap TA Yan L Cunningham D. Targeting the PI3K-AKT-mTOR signaling network in cancer. Chin J Cancer (2013) 32:253–65. 10.5732/cjc.013.10057 23642907 PMC3845556

[289] Lorusso PM. Inhibition of the PI3K/AKT/mTOR pathway in solid tumors. J Clin Oncol (2016) 34:3803–15. 10.1200/JCO.2014.59.0018 27621407 PMC6366304

[290] Gonzalez CD Alvarez S Ropolo A Rosenzvit C Bagnes MF Vaccaro MI. Autophagy, Warburg, and Warburg reverse effects in human cancer. Biomed Res Int (2014) 2014:926729. 10.1155/2014/926729 25197670 PMC4145381

[291] Song J Qu Z Guo X Zhao Q Zhao X Gao L Hypoxia-induced autophagy contributes to the chemoresistance of hepatocellular carcinoma cells. Autophagy (2009) 5:1131–44. 10.4161/auto.5.8.9996 19786832

[292] Huang Z Zhou L Chen Z Nice EC Huang C. Stress management by autophagy: implications for chemoresistance. Int J Cancer (2016) 139:23–32. 10.1002/ijc.29990 26757106

[293] Gozuacik D Akkoc Y Ozturk DG Kocak M. Autophagy-regulating microRNAs and cancer. Front Oncol (2017) 7:65. 10.3389/fonc.2017.00065 28459042 PMC5394422

[294] Bedi U Mishra VK Wasilewski D Scheel C Johnsen SA. Epigenetic plasticity: a central regulator of epithelial-to-mesenchymal transition in cancer. Oncotarget (2014) 5:2016–29. 10.18632/oncotarget.1875 24840099 PMC4039141

[295] Heery R Finn SP Cuffe S Gray SG Long non-coding RNAs: key regulators of epithelial-mesenchymal transition, tumour drug resistance and cancer stem cells. Cancers (Basel) (2017) 9:38–86. 10.3390/cancers9040038 PMC5406713 28430163

[296] Nurwidya F Takahashi F Murakami A Takahashi K. Epithelial mesenchymal transition in drug resistance and metastasis of lung cancer. Cancer Res Treat (2012) 44:151–6. 10.4143/crt.2012.44.3.151 23091440 PMC3467417

[297] Du B Shim JS. Targeting epithelial-mesenchymal transition (EMT) to overcome drug resistance in cancer. Molecules (2016) 21:965–80. 10.3390/molecules21070965 27455225 PMC6273543

[298] Zeng Y Wang T Liu Y Su Z Lu P Chen X LncRNA PVT1 as an effective biomarker for cancer diagnosis and detection based on transcriptome data and meta-analysis. Oncotarget (2017) 8:75455–66. 10.18632/oncotarget.20634 29088881 PMC5650436

[299] Reya T Morrison SJ Clarke MF Weissman IL. Stem cells, cancer, and cancer stem cells. Nature (2001) 414:105–11. 10.1038/35102167 11689955

[300] Ischenko I Seeliger H Kleespies A Angele MK Eichhorn ME Jauch KW Pancreatic cancer stem cells: new understanding of tumorigenesis, clinical implications. Langenbecks Arch Surg (2010) 395:1–10. 10.1007/s00423-009-0502-z 19421768

[301] Li G Liu C Yuan J Xiao X Tang N Hao J CD133(+) single cell-derived progenies of colorectal cancer cell line SW480 with different invasive and metastatic potential. Clin Exp Metastasis (2010) 27:517–27. 10.1007/s10585-010-9341-0 20617370

[302] Shankar S Nall D Tang SN Meeker D Passarini J Sharma J Resveratrol inhibits pancreatic cancer stem cell characteristics in human and KrasG12D transgenic mice by inhibiting pluripotency maintaining factors and epithelial-mesenchymal transition. PLoS One (2011) 6:e16530. 10.1371/journal.pone.0016530 21304978 PMC3031576

[303] Srivastava RK Tang SN Zhu W Meeker D Shankar S. Sulforaphane synergizes with quercetin to inhibit self-renewal capacity of pancreatic cancer stem cells. Front Biosci (Elite Ed) (2011) 3:515–28. 10.2741/e266 21196331 PMC4080794

[304] Nguyen LV Vanner R Dirks P Eaves CJ Cancer stem cells: an evolving concept. Nat Rev Cancer (2012) 12:133–43. 10.1038/nrc3184 22237392

[305] Pattabiraman DR Weinberg RA. Tackling the cancer stem cells – what challenges do they pose? Nat Rev Drug Discov (2014) 13:497–512. 10.1038/nrd4253 24981363 PMC4234172

[306] Tay Y Zhang J Thomson AM Lim B Rigoutsos I. MicroRNAs to Nanog, Oct4 and Sox2 coding regions modulate embryonic stem cell differentiation. Nature (2008) 455:1124–8. 10.1038/nature07299 18806776

[307] Liu C Tang DG. MicroRNA regulation of cancer stem cells. Cancer Res (2011) 71:5950–4. 10.1158/0008-5472.CAN-11-1035 21917736 PMC3177108

[308] Sun X Jiao X Pestell TG Fan C Qin S Mirabelli E MicroRNAs and cancer stem cells: the sword and the shield. Oncogene (2014) 33:4967–77. 10.1038/onc.2013.492 24240682

[309] Garg M. Emerging role of microRNAs in cancer stem cells: implications in cancer therapy. World J Stem Cells (2015) 7:1078–89. 10.4252/wjsc.v7.i8.1078 26435768 PMC4591786

[310] Han Y Yang YN Yuan HH Zhang TT Sui H Wei XL UCA1, a long non-coding RNA up-regulated in colorectal cancer influences cell proliferation, apoptosis and cell cycle distribution. Pathology (2014) 46:396–401. 10.1097/PAT.0000000000000125 24977734

[311] Li JY Ma X Zhang CB Overexpression of long non-coding RNA UCA1 predicts a poor prognosis in patients with esophageal squamous cell carcinoma. Int J Clin Exp Pathol (2014) 7:7938–44. 25550835 PMC4270573

[312] Wang F Ying HQ He BS Pan YQ Deng QW Sun HL Upregulated lncRNA-UCA1 contributes to progression of hepatocellular carcinoma through inhibition of miR-216b and activation of FGFR1/ERK signaling pathway. Oncotarget (2015) 6:7899–917. 10.18632/oncotarget.3219 25760077 PMC4480724

[313] Chen P Wan D Zheng D Zheng Q Wu F Zhi Q. Long non-coding RNA UCA1 promotes the tumorigenesis in pancreatic cancer. Biomed Pharmacother (2016) 83:1220–6. 10.1016/j.biopha.2016.08.041 27562722

[314] Wang H Guan Z He K Qian J Cao J Teng L. LncRNA UCA1 in anti-cancer drug resistance. Oncotarget (2017) 8:64638–50. 10.18632/oncotarget.18344 28969100 PMC5610032

[315] Jiao C Song Z Chen J Zhong J Cai W Tian S lncRNA-UCA1 enhances cell proliferation through functioning as a ceRNA of Sox4 in esophageal cancer. Oncol Rep (2016) 36:2960–6. 10.3892/or.2016.5121 27667646

[316] Taipale J Beachy PA. The Hedgehog and Wnt signalling pathways in cancer. Nature (2001) 411:349–54. 10.1038/35077219 11357142

[317] Fan Y Shen B Tan M Mu X Qin Y Zhang F Long non-coding RNA UCA1 increases chemoresistance of bladder cancer cells by regulating Wnt signaling. FEBS J (2014) 281:1750–8. 10.1111/febs.12737 24495014

[318] Xia P Xu XY. PI3K/Akt/mTOR signaling pathway in cancer stem cells: from basic research to clinical application. Am J Cancer Res (2015) 5:1602–9. 26175931 PMC4497429

[319] Safa AR. Resistance to cell death and its modulation in cancer stem cells. Crit Rev Oncog (2016) 21:203–19. 10.1615/CritRevOncog.2016016976 27915972 PMC5356509

[320] Li T Zheng Q An J Wu M Li H Gui X SET1A cooperates with CUDR to promote liver cancer growth and hepatocyte-like stem cell malignant transformation epigenetically. Mol Ther (2016) 24:261–75. 10.1038/mt.2015.208 26581161 PMC4817816

[321] Huang X Xiao R Pan S Yang X Yuan W Tu Z Uncovering the roles of long non-coding RNAs in cancer stem cells. J Hematol Oncol (2017) 10:62. 10.1186/s13045-017-0428-9 28245841 PMC5331729

[322] Zheng K Cubero FJ Nevzorova YA. c-MYC-making liver sick: role of c-MYC in hepatic cell function, homeostasis and disease. Genes (Basel) (2017) 8:123–43. 10.3390/genes8040123 28422055 PMC5406870

[323] Lin CP Liu JD Chow JM Liu CR Liu HE. Small-molecule c-Myc inhibitor, 10058-F4, inhibits proliferation, downregulates human telomerase reverse transcriptase and enhances chemosensitivity in human hepatocellular carcinoma cells. Anticancer Drugs (2007) 18:161–70. 10.1097/CAD.0b013e3280109424 17159602

[324] Pyndiah S Tanida S Ahmed KM Cassimere EK Choe C Sakamuro D. c-MYC suppresses BIN1 to release poly(ADP-ribose) polymerase 1: a mechanism by which cancer cells acquire cisplatin resistance. Sci Signal (2011) 4:ra19. 10.1126/scisignal.2001556 21447800

[325] Akita H Marquardt JU Durkin ME Kitade M Seo D Conner EA MYC activates stem-like cell potential in hepatocarcinoma by a p53-dependent mechanism. Cancer Res (2014) 74:5903–13. 10.1158/0008-5472.CAN-14-0527 25189530 PMC4199878

[326] Wu M Lin Z Li X Xin X An J Zheng Q HULC cooperates with MALAT1 to aggravate liver cancer stem cells growth through telomere repeat-binding factor 2. Sci Rep (2016) 6:36045 10.1038/srep36045 27782152 PMC5080550

[327] Chen FJ Sun M Li SQ Wu QQ Ji L Liu ZL Upregulation of the long non-coding RNA HOTAIR promotes esophageal squamous cell carcinoma metastasis and poor prognosis. Mol Carcinog (2013) 52:908–15. 10.1002/mc.21944 24151120

[328] Endo H Shiroki T Nakagawa T Yokoyama M Tamai K Yamanami H Enhanced expression of long non-coding RNA HOTAIR is associated with the development of gastric cancer. PLoS One (2013) 8:e77070. 10.1371/journal.pone.0077070 24130837 PMC3795022

[329] He Y Meng XM Huang C Wu BM Zhang L Lv XW Long noncoding RNAs: novel insights into hepatocelluar carcinoma. Cancer Lett (2014) 344:20–7. 10.1016/j.canlet.2013.10.021 24183851

[330] Mohamadkhani A. Long noncoding RNAs in interaction with RNA binding proteins in hepatocellular carcinoma. Hepat Mon (2014) 14:e18794. 10.5812/hepatmon.18794 24910706 PMC4030262

[331] Li H An J Wu M Zheng Q Gui X Li T LncRNA HOTAIR promotes human liver cancer stem cell malignant growth through downregulation of SETD2. Oncotarget (2015) 6:27847–64. 10.18632/oncotarget.4443 26172293 PMC4695030

[332] Yang Z Zhou L Wu LM Lai MC Xie HY Zhang F Overexpression of long non-coding RNA HOTAIR predicts tumor recurrence in hepatocellular carcinoma patients following liver transplantation. Ann Surg Oncol (2011) 18:1243–50. 10.1245/s10434-011-1581-y 21327457

[333] Ding C Cheng S Yang Z Lv Z Xiao H Du C Long non-coding RNA HOTAIR promotes cell migration and invasion via down-regulation of RNA binding motif protein 38 in hepatocellular carcinoma cells. Int J Mol Sci (2014) 15:4060–76. 10.3390/ijms15034060 24663081 PMC3975384

[334] Gupta RA Shah N Wang KC Kim J Horlings HM Wong DJ Long non-coding RNA HOTAIR reprograms chromatin state to promote cancer metastasis. Nature (2010) 464:1071–6. 10.1038/nature08975 20393566 PMC3049919

[335] Li F Mao G Tong D Huang J Gu L Yang W The histone mark H3K36me3 regulates human DNA mismatch repair through its interaction with MutSalpha. Cell (2013) 153:590–600. 10.1016/j.cell.2013.03.025 23622243 PMC3641580

[336] Pfister SX Ahrabi S Zalmas LP Sarkar S Aymard F Bachrati CZ SETD2-dependent histone H3K36 trimethylation is required for homologous recombination repair and genome stability. Cell Rep (2014) 7:2006–18. 10.1016/j.celrep.2014.05.026 24931610 PMC4074340

[337] Fan Y Shen B Tan M Mu X Qin Y Zhang F TGF-beta-induced upregulation of malat1 promotes bladder cancer metastasis by associating with suz12. Clin Cancer Res (2014) 20:1531–41. 10.1158/1078-0432.CCR-13-1455 24449823

[338] Cheng AS Lau SS Chen Y Kondo Y Li MS Feng H EZH2-mediated concordant repression of Wnt antagonists promotes beta-catenin-dependent hepatocarcinogenesis. Cancer Res (2011) 71:4028–39. 10.1158/0008-5472.CAN-10-3342 21512140

[339] Jonker DJ O’callaghan CJ Karapetis CS Zalcberg JR Tu D Au HJ Cetuximab for the treatment of colorectal cancer. N Engl J Med (2007) 357:2040–8. 10.1056/NEJMoa071834 18003960

[340] Weber J McCormack PL. Panitumumab: in metastatic colorectal cancer with wild-type KRAS. BioDrugs (2008) 22:403–11. 10.2165/0063030-200822060-00006 18998757

[341] Loupakis F Bria E Vaccaro V Cuppone F Milella M Carlini P Magnitude of benefit of the addition of bevacizumab to first-line chemotherapy for metastatic colorectal cancer: meta-analysis of randomized clinical trials. J Exp Clin Cancer Res (2010) 29:58. 10.1186/1756-9966-29-58 20504361 PMC2890550

[342] Roukos DH Targeting gastric cancer with trastuzumab: new clinical practice and innovative developments to overcome resistance. Ann Surg Oncol (2010) 17:14–7. 10.1245/s10434-009-0766-0 19841980 PMC2805793

[343] Grothey A Van Cutsem E Sobrero A Siena S Falcone A Ychou M Regorafenib monotherapy for previously treated metastatic colorectal cancer (CORRECT): an international, multicentre, randomised, placebo-controlled, phase 3 trial. Lancet (2013) 381:303–12. 10.1016/S0140-6736(12)61900-X 23177514

[344] Muro K Bang YJ Shankaran V Geva R Catenacci DVT Gupta S Relationship between PD-L1 expression and clinical outcomes in patients (Pts) with advanced gastric cancer treated with the anti-PD-1 monoclonal antibody pembrolizumab (Pembro; MK-3475) in KEYNOTE-012. J Clin Oncol (2015) 33:3 10.1200/jco.2015.33.3_suppl.3

[345] King J Palmer DH Johnson P Ross P Hubner RA Sumpter K Sorafenib for the treatment of advanced hepatocellular cancer – a UK audit. Clin Oncol (R Coll Radiol) (2017) 29:256–62. 10.1016/j.clon.2016.11.012 27964898

[346] Sui M Jiao A Zhai H Wang Y Wang Y Sun D Upregulation of miR-125b is associated with poor prognosis and trastuzumab resistance in HER2-positive gastric cancer. Exp Ther Med (2017) 14:657–63. 10.3892/etm.2017.4548 28672982 PMC5488498

[347] Kang YK Boku N Satoh T Ryu MH Chao Y Kato K Nivolumab in patients with advanced gastric or gastro-oesophageal junction cancer refractory to, or intolerant of, at least two previous chemotherapy regimens (ONO-4538-12, ATTRACTION-2): a randomised, double-blind, placebo-controlled, phase 3 trial. Lancet (2017) 390:2461–71. 10.1016/S0140-6736(17)31827-5 28993052

[348] Le DT Durham JN Smith KN Wang H Bartlett BR Aulakh LK Mismatch repair deficiency predicts response of solid tumors to PD-1 blockade. Science (2017) 357:409–13. 10.1126/science.aan6733 28596308 PMC5576142

[349] Overman MJ Lonardi S Wong KYM Lenz HJ Gelsomino F Aglietta M Durable clinical benefit with nivolumab plus ipilimumab in DNA mismatch repair-deficient/microsatellite instability-high metastatic colorectal cancer. J Clin Oncol (2018) 36:773–9. 10.1200/JCO.2017.76.9901 29355075

[350] Cortez MA Ivan C Valdecanas D Wang X Peltier HJ Ye Y PDL1 regulation by p53 via miR-34. J Natl Cancer Inst (2016) 108:djv303 10.1093/jnci/djv303 26577528 PMC4862407

[351] Xu S Tao Z Hai B Liang H Shi Y Wang T miR-424(322) reverses chemoresistance via T-cell immune response activation by blocking the PD-L1 immune checkpoint. Nat Commun (2016) 7:11406. 10.1038/ncomms11406 27147225 PMC4858750

[352] Smolle MA Calin HN Pichler M Calin GA. Noncoding RNAs and immune checkpoints-clinical implications as cancer therapeutics. FEBS J (2017) 284:1952–66. 10.1111/febs.14030 28132417

[353] Lin HM Mahon KL Spielman C Gurney H Mallesara G Stockler MR Phase 2 study of circulating microRNA biomarkers in castration-resistant prostate cancer. Br J Cancer (2017) 116:1002–11. 10.1038/bjc.2017.50 28278515 PMC5396108

[354] Gagez AL Duroux-Richard I Lepretre S Orsini-Piocelle F Letestu R De Guibert S miR-125b and miR-532-3p predict the efficiency of rituximab-mediated lymphodepletion in chronic lymphocytic leukemia patients. A French Innovative Leukemia Organization study. Haematologica (2017) 102:746–54. 10.3324/haematol.2016.153189 28126961 PMC5395115

[355] Wiemer EAC Wozniak A Burger H Smid M Floris G Nzokirantevye A Identification of microRNA biomarkers for response of advanced soft tissue sarcomas to eribulin: translational results of the EORTC 62052 trial. Eur J Cancer (2017) 75:33–40. 10.1016/j.ejca.2016.12.018 28214655

[356] Sclafani F Chau I Cunningham D Peckitt C Lampis A Hahne JC Prognostic role of the LCS6 KRAS variant in locally advanced rectal cancer: results of the EXPERT-C trial. Ann Oncol (2015) 26:1936–41. 10.1093/annonc/mdv285 26162609 PMC4551162

[357] Graziano F Canestrari E Loupakis F Ruzzo A Galluccio N Santini D Genetic modulation of the Let-7 microRNA binding to KRAS 3’-untranslated region and survival of metastatic colorectal cancer patients treated with salvage cetuximab-irinotecan. Pharmacogenomics J (2010) 10:458–64. 10.1038/tpj.2010.9 20177422

[358] Zhang W Winder T Ning Y Pohl A Yang D Kahn M A let-7 microRNA-binding site polymorphism in 3’-untranslated region of KRAS gene predicts response in wild-type KRAS patients with metastatic colorectal cancer treated with cetuximab monotherapy. Ann Oncol (2011) 22:104–9. 10.1093/annonc/mdq315 20603437 PMC8890483

[359] Sha D Lee AM Shi Q Alberts SR Sargent DJ Sinicrope FA Association study of the let-7 miRNA-complementary site variant in the 3’ untranslated region of the KRAS gene in stage III colon cancer (NCCTG N0147 Clinical Trial). Clin Cancer Res (2014) 20:3319–27. 10.1158/1078-0432.CCR-14-0069 24727325 PMC4084689

[360] Lin M Gu J Eng C Ellis LM Hildebrandt MA Lin J Genetic polymorphisms in microRNA-related genes as predictors of clinical outcomes in colorectal adenocarcinoma patients. Clin Cancer Res (2012) 18:3982–91. 10.1158/1078-0432.CCR-11-2951 22661538 PMC4141857

[361] Xing J Wan S Zhou F Qu F Li B Myers RE Genetic polymorphisms in pre-microRNA genes as prognostic markers of colorectal cancer. Cancer Epidemiol Biomarkers Prev (2012) 21:217–27. 10.1158/1055-9965.EPI-11-0624 22028396 PMC3253954

[362] Pardini B Rosa F Naccarati A Vymetalkova V Ye Y Wu X Polymorphisms in microRNA genes as predictors of clinical outcomes in colorectal cancer patients. Carcinogenesis (2015) 36:82–6. 10.1093/carcin/bgu224 25368035

[363] Sclafani F Chau I Cunningham D Lampis A Hahne JC Ghidini M Sequence variation in mature microRNA-608 and benefit from neo-adjuvant treatment in locally advanced rectal cancer patients. Carcinogenesis (2016) 37:852–7. 10.1093/carcin/bgw073 27381831 PMC5008250

[364] Laurent-Puig P Grisoni ML Heinemann V Bonnetain F Fontaine K Vazart C miR 31 3p as a predictive biomarker of cetuximab efficacy effect in metastatic colorectal cancer (mCRC) patients enrolled in FIRE-3 study. J Clin Oncol (2016) 34:3516–3516.

[365] Schultz NA Dehlendorff C Jensen BV Bjerregaard JK Nielsen KR Bojesen SE MicroRNA biomarkers in whole blood for detection of pancreatic cancer. JAMA (2014) 311:392–404. 10.1001/jama.2013.284664 24449318

[366] Shigeyasu K Toden S Zumwalt TJ Okugawa Y Goel A. Emerging role of microRNAs as liquid biopsy biomarkers in gastrointestinal cancers. Clin Cancer Res (2017) 23:2391–9. 10.1158/1078-0432.CCR-16-1676 28143873 PMC5433899

[367] Ozawa T Kandimalla R Gao F Nozawa H Hata K Nagata H A microRNA signature associated with metastasis of T1 colorectal cancers to lymph nodes. Gastroenterology (2018) 154:844–8.e7. 10.1053/j.gastro.2017.11.275 29199088 PMC5847452

[368] Hansen TF Carlsen AL Heegaard NH Sorensen FB Jakobsen A. Changes in circulating microRNA-126 during treatment with chemotherapy and bevacizumab predicts treatment response in patients with metastatic colorectal cancer. Br J Cancer (2015) 112:624–9. 10.1038/bjc.2014.652 25584492 PMC4333496

[369] Adams BD Parsons C Walker L Zhang WC Slack FJ. Targeting noncoding RNAs in disease. J Clin Invest (2017) 127:761–71. 10.1172/JCI84424 28248199 PMC5330746

[370] Rupaimoole R Slack FJ. MicroRNA therapeutics: towards a new era for the management of cancer and other diseases. Nat Rev Drug Discov (2017) 16:203–22. 10.1038/nrd.2016.246 28209991

[371] Janssen HL Reesink HW Lawitz EJ Zeuzem S Rodriguez-Torres M Patel K Treatment of HCV infection by targeting microRNA. N Engl J Med (2013) 368:1685–94. 10.1056/NEJMoa1209026 23534542

[372] Van Der Ree MH De Vree JM Stelma F Willemse S Van Der Valk M Rietdijk S Safety, tolerability, and antiviral effect of RG-101 in patients with chronic hepatitis C: a phase 1B, double-blind, randomised controlled trial. Lancet (2017) 389:709–17. 10.1016/S0140-6736(16)31715-9 28087069

[373] Van Zandwijk N Pavlakis N Kao SC Linton A Boyer MJ Clarke S Safety and activity of microRNA-loaded minicells in patients with recurrent malignant pleural mesothelioma: a first-in-man, phase 1, open-label, dose-escalation study. Lancet Oncol (2017) 18:1386–96. 10.1016/S1470-2045(17)30621-6 28870611

[374] Hsu SH Wang B Kota J Yu J Costinean S Kutay H Essential metabolic, anti-inflammatory, and anti-tumorigenic functions of miR-122 in liver. J Clin Invest (2012) 122:2871–83. 10.1172/JCI63539 22820288 PMC3408748

